# Proband-independent haplotyping based on NGS-based long-read sequencing for detecting pathogenic variant carrier status in preimplantation genetic testing for monogenic diseases

**DOI:** 10.3389/fmolb.2024.1329580

**Published:** 2024-03-07

**Authors:** Peiyu Zhang, Xiaomei Zhao, Qinshan Li, Yaqiong Xu, Zengmei Cheng, Lu Yang, Houmei Wang, Yang Tao, Guanyou Huang, Rui Wu, Hua Zhou, Shuyun Zhao

**Affiliations:** ^1^ Department of Obstetrics and Gynecology, Guizhou Medical University, Guiyang, China; ^2^ Reproductive Medicine Center, Department of Obstetrics and Gynecology of the Affiliated Hospital of Guizhou Medical University, Guiyang, China; ^3^ Department of Obstetrics and Gynecology, Affiliated Hospital of Guizhou Medical University, Guiyang, China; ^4^ Prenatal Diagnosis Center, Affiliated Hospital of Guizhou Medical University, Guiyang, China; ^5^ Reproductive Medicine Center, Department of Obstetrics and Gynecology of The First People’s Hospital of Bijie, Bijie, China

**Keywords:** monogenic genetic disease, proband-independent haplotyping based on long-read sequencing, preimplantation genetic testing for monogenic, incomplete families, *de novo* pathogenic variant

## Abstract

Preimplantation genetic testing for monogenic diseases (PGT-M) can be used to select embryos that do not develop disease phenotypes or carry disease-causing genes for implantation into the mother’s uterus, to block disease transmission to the offspring, and to increase the birth rate of healthy newborns. However, the traditional PGT-M technique has some limitations, such as its time consumption, experimental procedural complexity, and the need for a complete family or reference embryo to construct the haplotype. In this study, proband-independent haplotyping based on NGS-based long-read sequencing (Phbol-seq) was used to effectively construct haplotypes. By targeting the mutation sites of single gene disease point mutations and small fragment deletion carriers, embryos carrying parental disease-causing mutations were successfully identified by linkage analysis. The efficiency of embryo resolution was then verified by classical Sanger sequencing, and it was confirmed that the construction of haplotype and SNP linkage analysis by Phbol-seq could accurately and effectively detect whether embryos carried parental pathogenic mutations. After the embryos confirmed to be nonpathogenic by Phbol-seq-based PGT-M and confirmed to have normal copy number variation by Phbol-seq-based PGT-A were transplanted into the uterus, gene detection in amniotic fluid of the implanted embryos was performed, and the results confirmed that Phbol-seq technology could accurately distinguish normal genotype embryos from genetically modified carrier embryos. Our results suggest that Phbol-seq is an effective strategy for accurately locating mutation sites and accurately distinguishing between embryos that inherit disease-causing genes and normal embryos that do not. This is critical for Phbol-seq-based PGT-M and could help more single-gene disease carriers with incomplete families, *de novo* mutations or suspected germline mosaicism to have healthy babies with normal phenotypes. It also helps to reduce the transmission of monogenic genetic diseases in the population.

## 1 Introduction

Monogenic diseases are diseases caused by mutations in a single gene, either on one chromosome or in alleles on both homologous chromosomes. According to the Online Mendelian Inheritance in Man (OMIM) database, as of November 2020, more than 7,000 monogenic diseases have been discovered, of which nearly 6,000 have been identified in terms of phenotype and molecular pathogenic mechanisms (Wu et al., 2019). Although most single-gene disorders are rare, autosomal recessive (AR) diseases affect ∼1.7–5 in 1,000 neonates, autosomal dominant (AD) disorders affect 1.4 in 1,000 neonates, and single-gene diseases are responsible for a considerable disease burden (Wu et al., 2019; Xiao and Lauschke, 2021; Scriven, 2022). Most of the clinical manifestations of monogenic diseases are fatal, disabling, and stunting and are highly detrimental to health. Currently, only approximately 5% of single-gene disorders are treatable, and management is expensive; therefore, it is important to prevent or avoid the resulting birth defects (Cummings, 2018). Preimplantation genetic testing for monogenic/single-gene disorders (PGT-M) can be used to diagnose pathogenic gene variants in preimplantation embryos (Carvalho et al., 2020). The selection of embryos without the disease phenotype for transfer to the uterus is an effective measure to block transmission of the disease to offspring and has been used as the first reproductive option for couples at risk of having a child with a genetic disease.

PGT can be broadly divided into two groups. First-generation approaches involve targeted assessment of a gene or region coupled with nearby linked polymorphic markers to reduce errors associated with amplification bias. Such tests typically involve an educated guessing approach to selecting appropriate markers near the gene of interest, with luck playing a role in which markers are potentially informative. This sometimes leads to lengthy searches for flanking polymorphisms or even situations where it is not possible to flank the target or where markers are separated by distances that increase the risk of intervening recombination. Second-generation approaches involve high-density single-nucleotide polymorphism (SNP) arrays, in which a known location can be sandwiched between informative SNPs. While many arrays have hundreds of thousands of SNPs interrogated at a time, the number of ultimately informative SNPs may result in marker—target—marker gaps that have an increased incidence of meiotic recombination or areas of the genome, such as subtelomeric sites, that are not efficiently covered.

Traditionally, the approach to PGT-M has been to develop patient-specific assays, typically a composite of the mutation and nearby short tandem repeat (STR) markers, which are then used to directly analyze embryonic DNA for the causative parental single-gene mutation(s), with linked STRs enabling accurate embryo genotyping (Rechitsky et al., 2015). As an alternative indirect approach, SNP-based genome-wide karyomapping has been developed (Chang et al., 2023) to enable PGT-M to be successfully performed directly on embryonic multiple displacement amplification (MDA) products for almost any monogenic disease (Rogers et al., 2021; Ou et al., 2022). More recently, whole exome and whole genome haplotype sequencing methods have been shown to provide accurate embryo genotyping for PGT-M cases (Masset et al., 2019; Rogers et al., 2021). Even more recently, proof of concept for PGT-M in an alpha thalassemia deletion was demonstrated by direct third-generation sequencing of embryonic MDA products (Liu et al., 2021). In most cases, a family linkage must be established, which requires samples from relatives or a proband to identify the polymorphism patterns associated with the affected, carrier and noncarrier states. *De novo* mutations can often present a unique problem where there has been no prior segregation of markers in the proband and where linkage must be established after embryo creation and analysis (Yuan et al., 2020).

For most laboratories performing PCR- or NGS-based testing for PGT-M, comprehensive patient-specific workups are still needed to improve the efficiency and accuracy of embryo testing. Several researchers have proposed a comprehensive PGT method called HaploPGT, which combines reduced representation genome sequencing, read count analysis, B allele frequency and haplotyping analysis to simultaneously detect multiple genetic disorders in a single test (Xie et al., 2022). However, additional family members are needed to infer the parental haplotype to identify balanced translocations and monogenic mutations in the tested embryos. A recently described PGT protocol called OneGene PGT allows direct mutation testing, haplotyping and aneuploidy screening via next-generation sequencing (NGS) (Hornak et al., 2023). The whole-genome amplification product was combined with multiplex PCR for SNP enrichment. A dedicated bioinformatics tool allows mapping, genotype calling and haplotyping of informative SNP markers. This method has been implemented for several of the most common monogenic disorders. In particular, for some challenging PGT cases, the long-in-PacBio platform for molecular sequencing of parental carriers can be used as a direct means to rapidly infer mutations and proximal allele SNP profiles. The proband-independent haplotype analysis via the Phbol-seq method revealed high-quality long-segment sequences. A very narrow set of useful information heterozygous SNPs was identified with sufficient read segments containing target sequences to be phased with the target and, more importantly, to identify targets (mutations) for linkage confirmation and any other relevant structural changes in genes that may be important. Haplotypes were successfully constructed from SNP sites in the upper and lower 2 Mbp regions of the pathogenic locus. In a study of three couples carrying a single-gene disease, we found that evidence-independent haplotype analysis based on a parental carrier-based long-read sequencing (Phbol-seq) method identified a very close set of useful heterozygous SNPs that could be phased with the target without the need for other family members. Linkage information was used for NGS-based embryo testing in two couples who subsequently underwent a PGT-M cycle, culminating in a successful pregnancy with two euploid disease-free embryos.

## 2 Materials and methods

### 2.1 Patients

From April 2021 to March 2023, 3 couples who were admitted to the Affiliated Hospital of Guizhou Medical University for genetic counseling because one or both of them carried mutations for a single-gene disease were selected for PGT-M testing.

The inclusion criteria were as follows: 1. One or both spouses were carriers of a disease-causing/suspected disease-causing mutation for a monogenic disease; 2. A blastocyst could be formed after the ovulatory cycle. Regarding informed consent, this study was approved by the Reproductive Ethics Committee of the Affiliated Hospital of Guizhou Medical University. All couples received standardized genetic counseling before the start of the ovulation induction cycle, and all patients enrolled in the study gave informed consent and signed an informed consent form. All data used in this study were generated by the Phbol-seq-based PGT-M project at the Affiliated Hospital of Guizhou Medical University Centre for Reproduction. The data were coded, and the study was conducted in accordance with the tenets of the Declaration of Helsinki, good clinical practice and all applicable regulatory requirements.

The carrier (carrier 1) in family I was female and showed the following manifestations: low intelligence and cardiofacial syndrome type 3 (AD). Carrier 1 had the MAP2K1 gene:c.199G > A (heterozygote) mutation. Her husband had the wild-type MAP2K1 gene:c.199G > A. In family II, both the female (carrier 2) and male (carrier 3) had a mutation and had given birth to children with “heart disease,” which is part of the phenotype of patients with extremely long-chain acyl-CoA dehydrogenase deficiency caused by mutation of the ACADVL:c.1384-1387del (AR). In family III, there was a mutation in both the female (carrier 4: HBB gene:c.126_129del) and male (carrier 5: HBB gene (NM_000518.5):c.52A > T).

### 2.2 Preclinical PGT workup

#### 2.2.1 DNA extraction and quality control

The Nanobind CBB Big DNA Kit or Nanobind Tissue Big DNA Kit (Circulomics, NB-900-001-01) [High molecular weight DNA extraction kit (Bekon, China)] was used to extract genomic DNA from peripheral blood. The concentration of genomic DNA was determined using the Qubit^®^ dsDNA HS Assay Kit (Thermo Fisher Scientific, United States). The integrity of genomic DNA was tested for fragment size distribution using the gDNA 165 Kb Analysis Kit for FEMTO PULS system (FP-1002-0275) (Agilent, United States).

#### 2.2.2 Proband-independent haplotyping based on long-read sequencing (Phbol-seq) method

The qualified genomic DNA was diluted to a concentration of 1–3 ng/μL, 10 ng of the diluted genomic DNA was added to the fragmenting enzyme, and the DNA molecules introduced by the fragmenting enzyme were hybridized to magnetic beads with molecular tags to label the DNA molecules. In this case, the high-molecular-weight DNA molecules bound by the fragmenting enzyme were fragmented, and the fragmented DNA was labeled with molecular tags. The Phbol-seq library was generated by adding sequencing tags to both ends of the fragmented DNA molecule using DNA ligase. The library was enriched by PCR to match the amount of computer sequencing. The library was purified using DNA purification magnetic beads, and the purified products were quantified according to the Qubit^®^ dsDNA HS double-stranded DNA fluorescence quantitative kit instructions. The Agilent 2100 High Sensitivity DNA Kit (Agilent, United States) was used to determine the band size of the PCR products.

### 2.3 Bioinformatics analysis method

#### 2.3.1 Preprocessing of whole-genome sequencing data

According to the characteristics of the Phbol-seq label, the barcode molecular label sequence and the fixed sequence in the fastq file of reads were removed by the barcode table, and the barcode information was marked on the read name of the fastq file. Filtering of low-quality sequences for split fastq files. BWA software (v0.7.17-r1188) (Broad Institute, United States) was used to compare the fastq file with the reference genome (hg19) to generate the bam file. PCR weight removal and BQSR calibration were performed on the compared sequences (GATK v4.1.4.0-local). GATK software (Broad Institute, United States) was used to perform SNV analysis on the bam file after BQSR calibration and generate a vcf file.

#### 2.3.2 Genotype detection of PGT-M family carriers

For point mutations or small fragment insertions and deletions, the carrier could be identified by GATK software analysis results for the presence of gene variants, such as ACADVL c.1384_1387 del and HBB c.126_129del/c.52A > T. For long fragment deletion genotypes, the genotypes were confirmed by analyzing whether small fragments of copy number variation (CNV) were present in the gene region, and the breakpoint information of the CNV deletion was determined. The analysis of CNV was based on comparing the number of unique matches between the samples to be tested and the control samples in each window area, calculating the Log2RR value, finding the position of broken windows in the samples according to the circular binary segmentation (CBS) algorithm, merging the windows, and calculating the copy number of each area. According to the copy number results, it was determined whether each area is normal, duplicated or deleted.

#### 2.3.3 Haplotype of Phbol-seq-based PGT-M family carriers

Prior to typing, the bam and vcf files were split into 23 chromosome pairs. HapCut2 (v1.3.1) software (UC San Diego, USA) was used to assemble and heterozygous SNP/INDELs typing of target chromosomes based on the hg19 reference genome. For all chromosomes, the median N50 of assembled genome blocks were 10 Mbp. In a certain assembled block, barcode marking haplotype information (such as marking 1 or 0) was applied to each long fragment according to the assembly and SNP typing results. Taking HBB c.126_129del/c.52A > T mutation as example, this mutation was covered by several mapped reads with barcode information and marked haplotype information. For mutation base with covered reads with consistent marked haplotype information (for example 1), the marked haplotype information was signed with carrier haplotype. Wild type haplotype was signed based on the consistent marked haplotype information (for example 0) of wild type supported reads. Phased SNPs were extended based on the signed haplotypes in the assembled blocks.

#### 2.3.4 IGV map

The gene mutation map was constructed using IGV software (v2.11.6) (Broad Institute, USA).

## 3 Clinical PGT cycles

### 3.1 Embryo culture and biopsy

For families that proceeded with the clinical PGT cycle, embryo culture and biopsy procedures were performed per the standard clinical workflow of the Reproductive Medicine Center of the Affiliated Hospital of Guizhou Medical University. Briefly, oocytes were fertilized by intracytoplasmic sperm injection (ICSI) and cultured at 37°C in 5.5% CO_2_ and 5% O_2_ in single Qiunn’s^®^ medium (CooperSurgical, USA) under mineral oil. For TE biopsies, laser-assisted zona opening was performed on Days 5–7 postinsemination, at which time the biopsy was performed. Approximately 5–10 cells were aspirated with laser pulses (Saturn 5TM Laser system) in combination with mechanical detachment (flicking). All embryo biopsies were washed in 0.1% PVP/PBS droplets before transferring them into PCR tubes filled with 2 μL PBS. All embryo biopsies were stored at −20°C until further processing.

### 3.2 Embryo biopsy processing

All biopsies were whole-genome amplified using multiple displacement amplification (MDA) (REPLI-g Single Cell kit, Qiagen, Germany). All steps were performed according to the manufacturer’s protocol. Amplified biopsies were quantified using a QubitTM dsDNA BR Assay Kit.

### 3.3 Integrated phbol-seq-based PGT detection analysis

Testing for aneuploidy, disease-causing genes and runs of homozygosity was performed by integrated PGT detection analysis as described by Zhang et al. (2021).

#### 3.3.1 Whole-genome sequencing data Preprocessing

The data format for the sample sequencing data was the Fastq format, and the data in the Fastq file were compared to the reference genome (hg19) using BWA software (v0.7.17-r1188) (Broad Institute, United States) to generate the bam file. PCR weight removal (Picard v 2.18.9-SNAPSHOT, Broad Institute, United States) and BQSR calibration (GATK v4.1.4.0-local) were performed on the compared sequences. GTAK software was used to analyze the mutation and generate the vcf file.

#### 3.3.2 CNV analysis

The number of unique matches between the samples to be tested and the control samples in each window area was compared, and the Log2RR value was calculated. The circular binary segmentation (CBS) algorithm was used to find the location of broken windows in the samples, and the windows were merged to calculate the copy number of each area. The copy number results indicated whether each area was normal, duplicated, deleted and chimeric duplicated or deleted.

#### 3.3.3 Z score analysis

Conventional CNV analysis was combined with SNP analysis to calculate the BAF distribution and the proportion of heterozygous SNPs, to calculate the Z score of each chromosome, to distinguish the presence of multiple sets of genetic material in the test sample by outliers, and to distinguish the cases of haploids (Z value ≥ 3).

#### 3.3.4 Runs of homozygosity (ROH) analysis

The ROH region is homozygous, which is obviously different from the diploid region, which contains many heterozygous sites. According to this characteristic, the genome was divided into several windows, and the proportion of heterozygous sites for F1 in each window was calculated and compared with the proportion of heterozygous sites for F2 in the corresponding region of the reference sample. The log (F1/F2) value was calculated. In the absence of CNV, log (F1/F2)≤-1 indicates that there are fewer heterozygous sites in that region, which may be the ROH region.

## 4 TE biopsy direct mutation analysis using Sanger sequencing

For a subset of TE biopsies that were processed using integrated Phbol-seq-based PGT, direct mutation analysis coupled with targeted long-read amplicon sequencing (LRS) haplotyping was also performed. Whole-genome amplified embryo biopsies, as well as unaffected partners, were subjected to Sanger sequencing and data analysis using the preclinical PGT workflow described above.

## 5 Prenatal follow-up of embryo transfers

Patients with persistent pregnancies after embryo transfer underwent prenatal diagnosis to verify the Phbol-seq-based PGT results. Amniocentesis was performed at 18–20 weeks gestation, and amniotic cells were purified for fetal chromosome analysis. Secondary amniotic cells were karyotyped, parental mutations were detected by amniotic cell DNA Sanger sequencing, and pathogenic microdeletions and microduplications were detected by amniotic cell DNA copy number variation sequencing.

## 6 Results

### 6.1 Phbol-seq-based preclinical PGT workup identifies parental origin of the mutated allele

**FIGURE 1:**
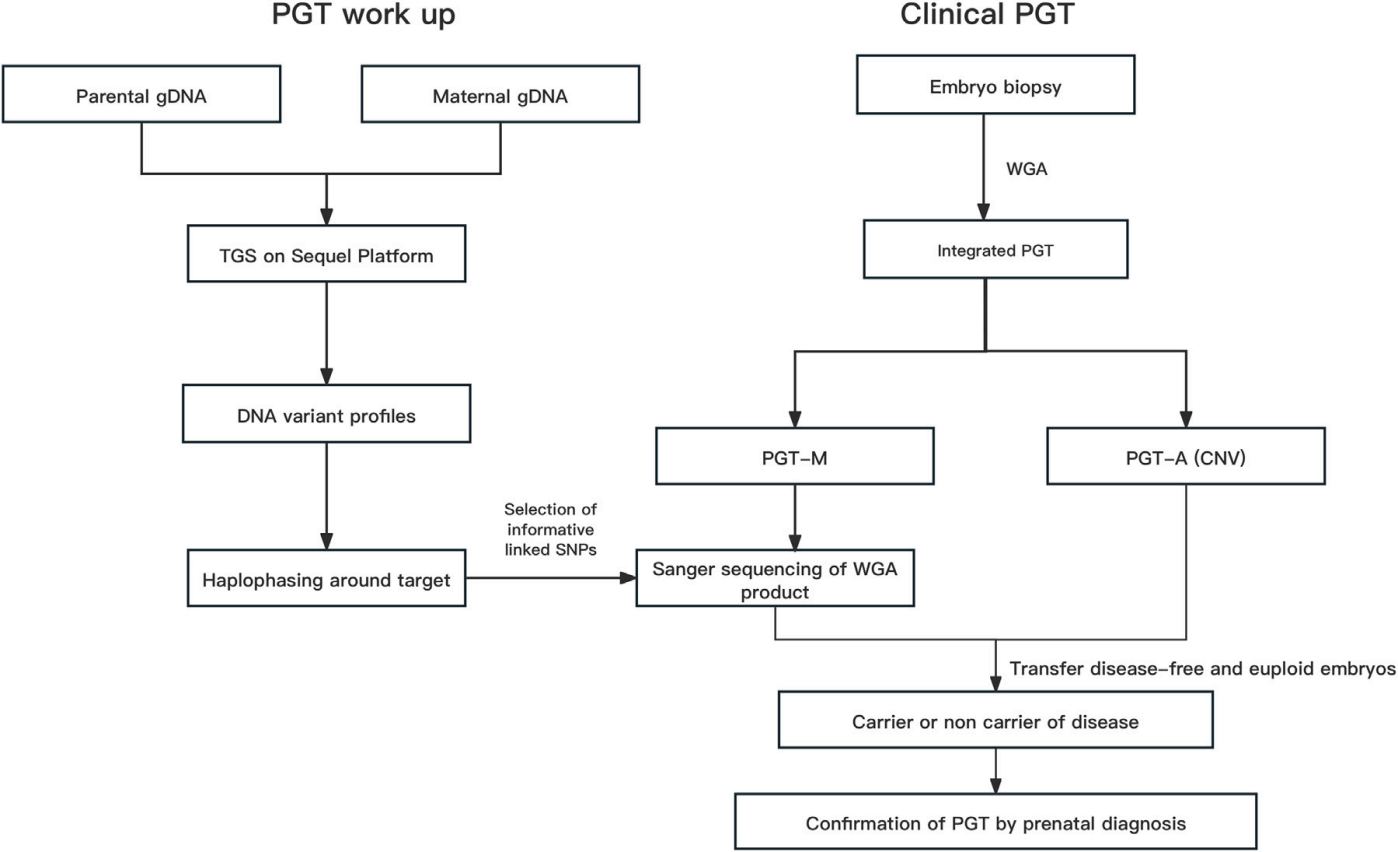
The overall strategy for clinical PGT Abbreviations: gDNA, genomic DNA; Hap, haplotype; PGT, preimplantation genetic testing; Integrated PGT, integrated preimplantation genetic testing; PGT-A, preimplantation genetic testing for aneuploidy; PGT-M, preimplantation genetic testing for monogenic diseases; Phbol-seq, proband-independent haplotyping based on long-read sequencing; WGA, whole-genome amplification.

The overall strategy for clinical PGT is shown in Figure 1. All parental gDNA samples for the 3 single-gene cases were successfully sequenced on the PacBio platform, providing high-quality long-read sequences (The quality control data is shown in Table 1). For all samples, there was high genome coverage (93.36%–96.54%), with long read lengths (36.9–48.8 kb). Importantly, there were sufficient reads containing the target sequence to identify precise mutations. The patients who underwent Phbol-seq-based PGT-M had autosomal dominant genetic disease, namely, cardio-facio-cutaneous syndrome 3 involving the MAP2K1 gene; autosomal recessive genetic disease, namely, very-long chain acyl-CoA dehydrogenase deficiency (VLCAD) involving the ACADVL gene; or autosomal recessive genetic disease, namely, b-thalassemia involving the HBB gene. Among them, multiple overlapping reads harboring pathogenic parental carrier mutations were identified (Table 2). The pathogenic site of carrier 1 (maternal) at chr15:66727483 was identified by long fragment sequencing. The pathogenic sites of carrier 2 (maternal) and carrier 3 (paternal) were located at chr17:7127338-7127341. The pathogenic site of carrier 4 (maternal) was located at chr11:5247993-5247996, and the haplotype of carrier 5 (paternal) was located at chr11:5248200. At the same time, the haplotype was successfully constructed from SNP sites in the upper and lower 2 Mbp range of the pathogenic sites (The number of effective phasing SNPs and the distance to the target genes are described in Table 3).

**TABLE 1:** Quality control data for Phbol-seq sequencing of parental carrier samples.

PGT case	Total bases sequenced	Effective reads	Effective coverage	Q30%	Mean DNA fragment length (bp)	Mapped sequencing reads	Read mapping rate (%)	Coverage >10X rate (%)	Mutation position reads	Haplotype block size (Mb)
Carrier 1	84,406,740,000	765,245,910	25.51	89.19	48,809	764,513,124	99.9	96.54	17	>2
Carrier 2	79,662,837,000	710,706,130	23.69	87.82	44,790	710,036,505	99.91	95.60	15	>2
Carrier 3	74,449,852,000	665,000,796	22.17	87.01	41,338	663,962,015	99.84	93.36	16	>2
Carrier 4	71,614,676,000	640,093,652	21.34	87.24	36,948	639,422,654	99.9	94.29	18	>2
Carrier 5	78,560,363,000	710,356,530	23.68	87.57	37,490	709,414,889	99.85	94.78	19	>2

Notes:

Effective reads: clean reads with pair-end matched DNA, molecular barcodes. Effective coverage: total genome coverage by effective reads.

Mean DNA, fragment length: reads sharing the same barcode in a certain region of a chromosome are defined with a DNA, fragment.

**TABLE 2:** Mapping of mutation targets.

Genetic lesion detected by molecular testing for each PGT case	Mutation position (hg19)	No. of reads spanning mutation
MI		
MAP2K1 gene: c.199G>A (Mat)	chr15:66727483	17/15
MII		
ACADVL gene: c.1384_1387del (Mat)	chr17:7127338–7127341	16
ACADVL gene: c.1384_1387del (Pat)	chr17:7127338–7127341	20
MIII		
HBB gene: c.126_129del (Mat)	chr11:5247993–5247996	18
HBB gene: c.52A>T (Pat)	chr11:5248200	19

Abbreviations: Mat, maternal; Pat: paternal; PGT, preimplantation genetic testing.

**TABLE 3:** The number of effective phasing SNPs and the distance to the target genes.

PGT case	Mutations	Selective SNPs for embryo phasing (upstream/downstream)	Distance from targeted genes (upstream/downstream)
MI	Maternal carrier of MAP2K1 gene: c.199G>A (Heterozygous)	29/33	1,934.95 Kb/829.86 Kb
MII	Maternal carrier of ACADVL gene: c.1384_1387del (Heterozygous); Paternal carrier of ACADVL gene: c.1384_1387del (Heterozygous)	25/31 (Maternal)	1,804.21 Kb/1,970.82 Kb (Maternal)
		15/19 (Paternal)	1,476.59 Kb/1,676.78 Kb (Paternal)
MIII	Maternal carrier of HBB gene: c.126_129del (Heterozygous); Paternal carrier of HBB gene: c.52A>T (Heterozygous)	241/151 (Maternal)	1,994.72 Kb/1,998.2 Kb (Maternal)
		169/146 (Paternal)	1,981.02 Kb/1,908.13 Kb (Paternal)

Note: For HBB gene mutations, upstream or downstream genome regions acquired more selective SNPs as a result of relative higher minor allele frequencies.

### 6.2 Embryo analysis results by phbol-seq-based integrated preimplantation genetic testing: pathogenic variation results and CNV results

**FIGURE 2:**
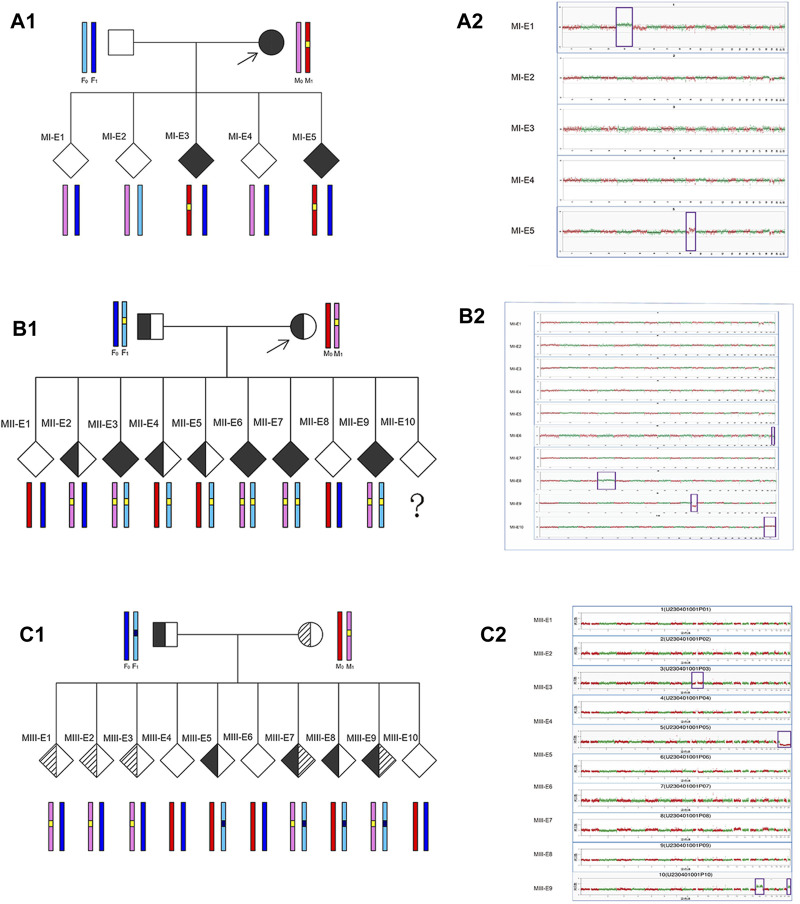
Embryo analysis results by Phbol-seq-based integrated preimplantation genetic testing Preclinical PGT workup and clinical PGT cycle in families with monogenic diseases. For each embryo, parental haplotype blocks are depicted: light/dark blue and pink/red haplotypes represent paternal and maternal alleles, respectively. In chromosome plots from Phbol-seq-based PGT-A data, the y-axis indicates the copy number, and the x-axis indicates the autosome number. Red and green dots indicate the copy numbers of different chromosomes for each embryo. Each point is at 1 Mb resolution. Abnormal copy number variation is marked by purple boxes. **(A1)** Preclinical PGT workup is shown for family I (MI) with a *de novo* pathogenic variant in the MAP2K1 gene in the mother. The results of Phbol-seq-based PGT-M using the affected mother as the phasing reference. The yellow line indicates the region of interest on chromosome 15. Based on the inheritance of iSNVs, two embryos (MI-E3 and MI-E5) inherited the risk allele from the mother (red haplotype) and the mutation (yellow line), and three embryos (MI-E1, MI-E2, and MI-E4) inherited the nonrisk allele from the mother (pink haplotype). **(A2)** Results from the five-embryo analysis of Phbol-seq-based PGT-A (copy number variation). Three embryos (MI-E2, MI-E3 and MI-E4) are indicated euploid, while embryo MI-E1 is indicated trisomy 4, and embryo MI-E5 is indicated dup (9) (q21.11q21.2) [GRCh37/hg19] (70960001–79700000) ×3; dup (mosaic) (9) (q21.2q34.3) [GRCh37/hg19] (79700001–141213431) ×3 (45%). **(B1)** Preclinical PGT workup is shown for family II (MII) with a *de novo* pathogenic variant in the ACADVL gene in the mother and father. The results of Phbol-seq-based PGT-M using affected mother and father as the phasing reference. The yellow line indicates the region of interest on chromosome 17. Based on the inheritance of iSNVs, four embryos (MII-E3, MII-E6, MII-E7, and MII-E9) inherited the risk allele and the mutation (yellow line) from both the mother (pink haplotype) and the father (light blue haplotype); two embryos (MII-E1 and MII-E8) inherited the nonrisk allele from both the mother (red haplotype) and father (dark blue haplotype); one embryo (MII-E2) inherited the risk allele only from the mother; two embryos (MII-E4 and MII-E5) inherited the risk allele only from the father; Embryo MII-E10 was a phasing failure resulting from abnormal copy number variation (69, XNN). **(B2)** Ten embryo analysis results of Phbol-seq-based PGT-A. Six embryos (MII-E1, MII-E2, MII-E3, MII-E4, MII-E5, and MII-E10) were indicated as euploid; however, embryo MII-E10 was indicated as having abnormal copy number variation (69, XNN). Three embryos also had abnormal copy number variation (MII-E6: -(mosaic) (22) (30%); MII-E8: +(mosaic) (4) (55%); MII-E9: del (11) (p15.5p11.2) [GRCh37/hg19](1240001–48520000)×1). **(C1)** Preclinical PGT workup is shown for family III (MIII) with a *de novo* pathogenic variant in the HBB gene in the mother (c.126_129del) and father (c.52A>T). The results of Phbol-seq-based PGT-M using the affected mother and father as the phasing reference. The black line indicates the region of interest on chromosome 11 from the father, and the yellow line indicates the region of interest on chromosome 11 from the mother. Based on the inheritance of iSNVs, two embryos (MIII-E7 and MIII-E9) inherited the risk allele and the mutation from both the mother (pink haplotype) and the father (light blue haplotype); three embryos (MIII-E4, MIII-E6 and MIII-E10) inherited the nonrisk allele from both the mother (red haplotype) and father (dark blue haplotype); three embryos (MIII-E1, MIII-E2, and MIII-E3) inherited the risk allele only from the mother; and two embryos (MIII-E5 and MIII-E8) inherited the risk allele only from the father. **(C2)** Ten embryo analysis results of Phbol-seq-based PGT-A. Seven embryos (MIII-E1, MIII-E2, MIII-E4, MIII-E6, MIII-E7, MIII-E8, and MIII-E9) are indicated euploid. Three embryos had abnormal copy number variation (MIII-E3: dup (mosaic) (9) (p13.1q21.11). seq [GRCh37/hg19] (39010001-71860000) ×3 (47%); MIII-E5: −X; MIII-E10: +16; +22). iSNVs: informative single-nucleotide variants; del: deletion; dup: duplication.

Based on the results of the preclinical PGT examination, the 3 analyzed couples each successfully underwent 3 ICSI-PGT cycles (an average of 1 cycle per couple with 25/3 embryos per cycle). All embryos were analyzed by Phbol-seq-based integrated PGT. A total of 25 embryos were analyzed, and the results are presented in Table 4. There were 8 disease-free (unaffected) embryos, 8 carrier embryos, 8 affected embryos and 1 embryo with chromosome 69, XNN, wherein it was impossible to distinguish haplotypes. Of the 16 unaffected or carrier embryos, 11 had chromosomal euploidy, 3 had chromosomal aneuploidy, and 2 had chromosomal mosaicism. Specifically, in family I, embryos MI-E1, MI-E2, and MI-E4 did not carry disease-causing mutations, whereas embryos MI-E3 and MI-E5 carried disease-causing mutations found in the female (carrier 1) (Figure 2A1). In family II, embryos MII-E1 and MII-E8 did not carry conjugal disease-causing variants; embryos MII-E3, MII-E6, MII-E7 and MII-E9 carried conjugal disease-causing variants; embryo MII-E2 carried the female (carrier 2) disease-causing variants; and embryos MII-E4 and MII-E5 carried the male variant (carrier 3) (Figure 2B1). In family III, embryos MIII-E4, MIII-E6 and MIII-E10 did not carry bilateral disease-causing variants; embryos MIII-E1, MIII-E2, MIII-E3, MIII-E7 and MIII-E9 carried only the female (carrier 4) disease-causing variants; and embryos MIII-E5 and MIII-E8 carried only the male (carrier 5) disease-causing variants (Figure 2C1). The results show that Phbol-seq-based sequencing could accurately identify the mutation sites in the samples and be used to distinguish whether the embryos carried disease-causing mutations in the PGT-M cycle.

**TABLE 4:** The mutation and copy number variation results analyzed by Phbol-seq of embryos.

Carrier status for each PGT case	Embryo	PGT results (pathogenic variation results)	PGT results (CNV results)
MI			
Maternal carrier of MAP2K1 gene: c.199G>A (Heterozygous)	MI-E1	MAP2K1 c.199G>A (WT)	+4
	MI-E2	MAP2K1 c.199G>A (WT)	Euploid
	MI-E3	MAP2K1 c.199G>A (Het, Mat)	Euploid
	MI-E4	MAP2K1 c.199G>A (WT)	Euploid
	MI-E5	MAP2K1 c.199G>A (Het, Mat)	dup (9)(q21.11q21.2)[GRCh37/hg19](70960001-79700000)×3; dup (mosaic)(9)(q21.2q34.3)[GRCh37/hg19] (79700001-141213431)×3 (45%)
MII			
Maternal carrier of ACADVL gene: c.1384_1387del (Heterozygous); Paternal carrier of ACADVL gene: c.1384_1387del (Heterozygous)	MII-E1	ACADVL c.1384_1387del (Hom, Mat and Pat)	Euploid
	MII-E2	ACADVL c.1384_1387del (Het, Mat)	Euploid
	MII-E3	ACADVL c.1384_1387del (Hom)	Euploid
	MII-E4	ACADVL c.1384_1387del (Het, Pat)	Euploid
	MII-E5	ACADVL c.1384_1387del (Het, Pat)	Euploid
	MII-E6	ACADVL c.1384_1387del (Hom)	−(mosaic)(22)(30%)
	MII-E7	ACADVL c.1384_1387del (Hom)	Euploid
	MII-E8	ACADVL c.1384_1387del (WT)	+(mosaic)(4)(55%)
	MII-E9	ACADVL c.1384_1387del (Hom)	del (11)(p15.5p11.2)[GRCh37/hg19](1240001-48520000)×1
	MII-E10	-	69,XNN
MIII			
Maternal carrier of HBB gene: c.126_129del (Heterozygous); Paternal carrier of HBB gene: c.52A>T (Heterozygous)	MIII-E1	β^CD41-42^/β^N^	Euploid
	MIII-E2	β^CD41-42^/β^N^	Euploid
	MIII-E3	β^CD41-42^/β^N^	dup (mosaic)(9)(p13.1q21.11).seq [GRCh37/hg19](39010001-71860000)×3 (47%)
	MIII-E4	β^N^/β^N^	Euploid
	MIII-E5	β^CD17^β^N^	−X
	MIII-E6	β^N^/β^N^	Euploid
	MIII-E7	β^CD41-42^/β^CD17^	Euploid
	MIII-E8	β^CD17^β^N^	Euploid
	MIII-E9	β^CD41-42^/β^CD17^	Euploid
	MIII-E10	β^N^/β^N^	+16; +22

Abbreviations: PGT, preimplantation genetic testing.

Chromosome analysis was performed on five embryos (MI-E1-MI-E5) from family I by Phbol-seq-based PGT-A. Embryos MI-E2, MI-E3 and MI-E4 were euploid, but embryos MI-E1 and MI-5 had abnormal copy number variation (Figure 2A2; Table 4).

Chromosome analysis of 10 embryos (MII-E1-MII-E10) from family II by Phbol-seq-based PGT-A showed that embryos MII-E1, MII-E2, MII-E3, MII-E4, MII-E5, and MII-E7 were euploid. However, embryos MII-E6, MII-E8, MII-E9, and MII-E10 had abnormal copy number variation according to Phbol-seq-based PGT-A (Figure 2B2; Table 4). The chromosome analysis performed on 10 embryos from family III (MIII-E1-MIII-E10) showed that embryos MIII-E1, MIII-E2, MIII-E4, MIII-E6, MIII-E7, MIII-E8, and MIII-E9 were euploid and that embryos MIII-E3, MIII-E5, and MIII-E10 had abnormal copy number variation according to Phbol-seq-based PGT-A (Figure 2C2; Table 4).

Based on the whole genome information of the unaffected embryos, further transfer sequencing was performed according to internal guidelines (Dimitriadou et al., 2017). The final embryo MI-E4 (morphological rating 4BB) and embryo MII-E1 (morphological rating 5BC) were transferred to the maternal uterus with informed consent. Both embryos were successfully implanted. At 18–20 weeks of gestation, amniotic fluid samples were taken for genetic testing, and the genetic results were completely normal. Prenatal diagnosis results showed that the Phbol-seq-based PGT-M results were correct. There are currently 2 cases of delivery. All children born were free of disease. Family III is currently undergoing endometrial preparation for embryo transfer.

### 6.3 Sanger sequencing verification

**FIGURE 3:**
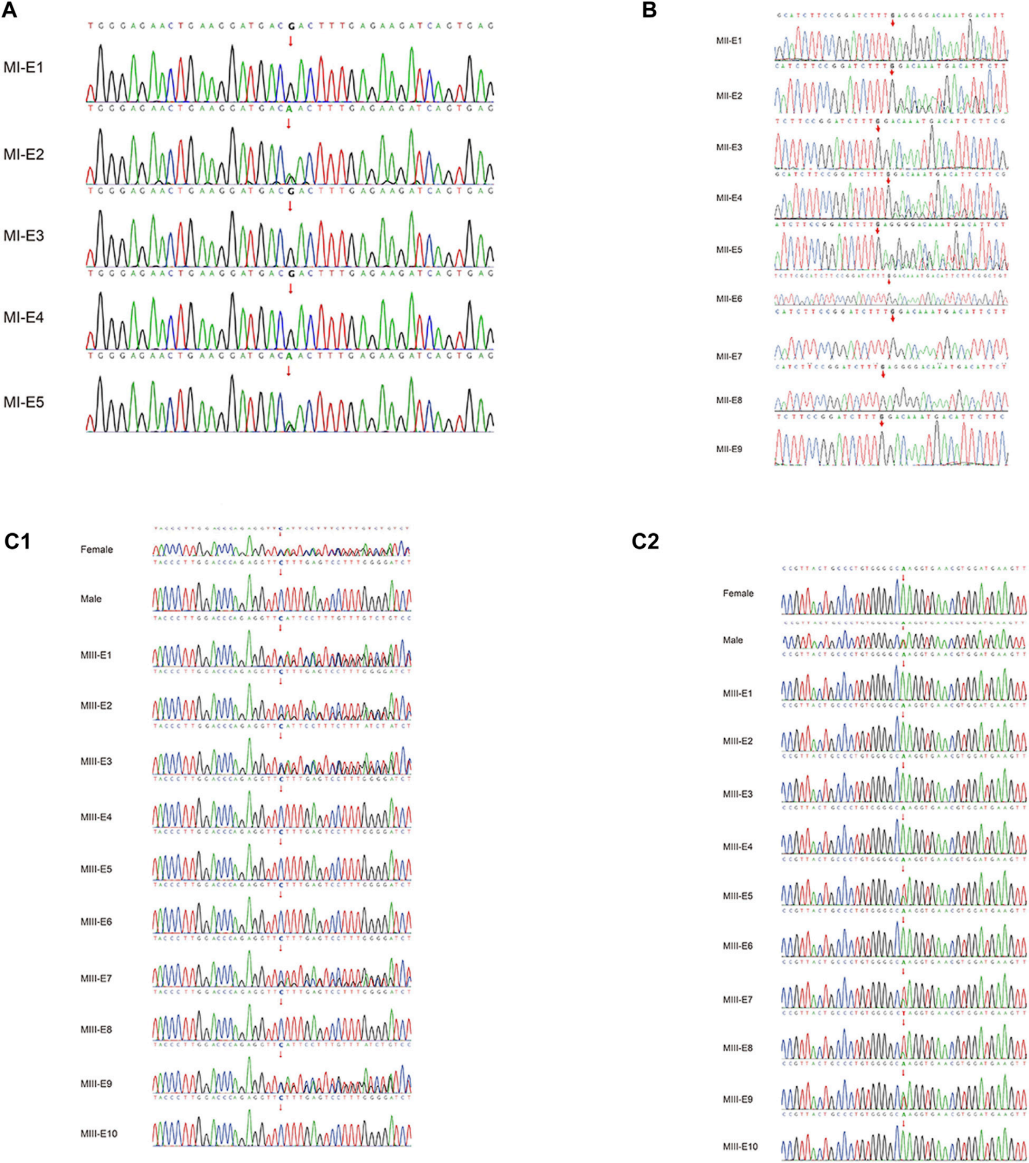
Pathogenic variant verification in embryos by Sanger sequencing. Allelic haplotype mapping to identify the carrier status of the embryos from families. The pathogenic variants in genes identified by Sanger sequencing analysis of the related embryos. **(A)** In family I, two embryos (MI-E3 and MI-E5) inherited the MAP2K1 gene c.199G>A, while three embryos (MI-E1, MI-E2 and MI-E4) did not inherit the mutation from the mother. **(B)** In family II, four embryos (MII-E3, MII-E6, MII-E7 and MII-E9) inherited the ACADVL gene:c.1384_1387del mutation from both the mother and the father; two embryos (MII-E1 and MII-E8) did not inherit the mutation from either the mother or the father; one embryo (MII-E2) inherited the mutation only from the mother; two embryos (MII-E4 and MII-E5) inherited the mutation only from the father; embryo MII-E10 was a phasing failure resulting from abnormal copy number variation (69,XNN). **(C1)** In family III, five embryos (MIII-E1, MIII-E2 MIII-E3, MIII-E7 and MIII-E9) inherited the HBB gene: c.126_129del mutation from the mother; five embryos (MIII-E4, MIII-E5, MIII-E6, MIII-E8 and MIII-E10) did not inherit the mutation from the mother. **(C2)** In family III, four embryos (MIII-E5, MIII-E7, MIII-E8 and MIII-E9) inherited the HBB gene:c.52A>T from the father; six embryos (MIII-E1, MIII-E2, MIII-E3, MIII-E4, MIII-E6, MIII-E8 and MIII-E10) did not inherit the mutation from the father. The red line indicates nucleotide T, the green line indicates nucleotide A, the blue line indicates nucleotide C, and the black line indicates nucleotide G.

All samples from the families were evaluated by Sanger sequencing, and the results were found to be consistent with the results of Phbol-seq-based PGT-M regarding the pathogenic gene carrier status of each embryo (Figure 3; Table 5). Detailed SNPs selected for haplotyping in the prospective parents and embryos are shown in Supplementary Tables S1.1–1.3.

**TABLE 5:** The concordance of Sanger sequencing for each of the whole biopsied embryos with previous Phbol-seq-based PGT results obtained from biopsied samples.

Sanger results (carrier) for each PGT case	Embryo	PGT results	Sanger results (biopsied embryo)	Concordance between PGT and sanger
MI				
Carrier 1: MAP2K1 gene:c.199G>A (Heterozygous)	MI-E1	MAP2K1 c.199G>A (WT)	MAP2K1 c.199G>A (WT)	Concordant
	MI-E2	MAP2K1 c.199G>A (WT)	MAP2K1 c.199G>A (WT)	Concordant
	MI-E3	MAP2K1 c.199G>A (Het, Mat)	MAP2K1 c.199G>A (Het, Mat)	Concordant
	MI-E4	MAP2K1 c.199G>A (WT)	MAP2K1 c.199G>A (WT)	Concordant
	MI-E5	MAP2K1 c.199G>A (Het, Mat)	MAP2K1 c.199G>A (Het, Mat)	Concordant
MII				
Carrier 2: ACADVL gene:c.1384_1387del (Heterozygous)	MII-E1	ACADVLc.1384_1387del (WT)	ACADVLc.1384_1387del (WT)	Concordant
	MII-E2	ACADVLc.1384_1387del (Het, Mat)	ACADVLc.1384_1387del (Het, Mat)	Concordant
	MII-E3	ACADVLc.1384_1387del (Hom)	ACADVLc.1384_1387del (Hom)	Concordant
	MII-E4	ACADVLc.1384_1387del (Het, Pat)	ACADVLc.1384_1387del (Het, Pat)	Concordant
	MII-E5	ACADVLc.1384_1387del (Het, Pat)	ACADVLc.1384_1387del (Het, Pat)	Concordant
	MII-E6	ACADVLc.1384_1387del (Hom)	ACADVLc.1384_1387del (Hom)	Concordant
	MII-E7	ACADVLc.1384_1387del (Hom)	ACADVLc.1384_1387del (Hom)	Concordant
Carrier 3: ACADVL gene:c.1384_1387del (Heterozygous)	MII-E8	ACADVLc.1384_1387del (WT)	ACADVLc.1384_1387del (WT)	Concordant
	MII-E9	ACADVLc.1384_1387del (Hom)	ACADVLc.1384_1387del (Hom)	Concordant
	MII-E10	-	-	
MIII				
Carrier 4: Female HBB gene: c.126_129del (β^CD41-42^) (Heterozygous)	MIII-E1	β^CD41-42^/β^N^	β^CD41-42^/β^N^	Concordant
	MIII-E2	β^CD41-42^/β^N^	β^CD41-42^/β^N^	Concordant
	MIII-E3	β^CD41-42^/β^N^	β^CD41-42^/β^N^	Concordant
	MIII-E4	β^N^/β^N^	β^N^/β^N^	Concordant
Carrier 5: Male HBB gene: c.52A>T (β^CD17^) (Heterozygous)	MIII-E5	β^CD17^β^N^	β^CD17^β^N^	Concordant
	MIII-E6	β^N^/β^N^	β^N^/β^N^	Concordant
	MIII-E7	β^CD41-42^/β^CD17^	β^CD41-42^/β^CD17^	Concordant
	MIII-E8	β^CD17^β^N^	β^CD17^β^N^	Concordant
	MIII-E9	β^CD41-42^/β^CD17^	β^CD41-42^/β^CD17^	Concordant
	MIII-E10	β^N^/β^N^	β^N^/β^N^	Concordant

Abbreviations: PGT, preimplantation genetic testing.

### 6.4 Prenatal follow-up of embryo transfers

**FIGURE 4:**
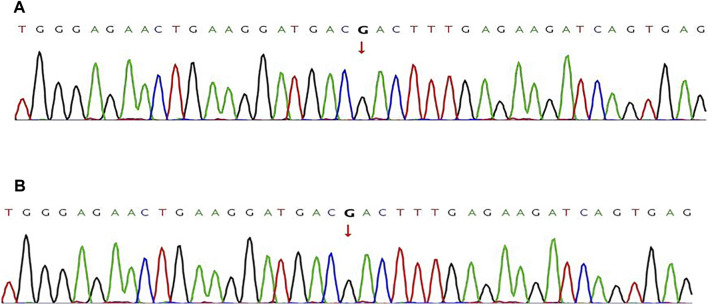
Pathogenic variant verification in fetal amniotic cells by Sanger sequencing Sanger sequencing was used to detect the pathogenic variants in fetal samples directly. **(A)** the wild-type MAP2K1 gene:c.199G>A of the fetus from family I. **(B)** The wild-type ACADVL gene: c.1384_1387del of the fetus from family II. The red line indicates nucleotide T, the green line indicates nucleotide A, the blue line indicates nucleotide C, and the black line indicates nucleotide G.

Sanger sequencing was used to evaluate cells obtained from the amniotic fluid of two pregnant women, and it was found that the fetal amniotic cell verification results by Sanger sequencing were consistent with the results by Phbol-seq-PGT-M regarding the pathogenic gene carrier status of each embryo (Figure 4). NGS was used to analyze the copy number variation in the fetal amniotic cells, and the results showed that the two fetuses were euploid (Supplementary Figure S1).

## 7 Discussion

Improvements in accuracy and throughput have the potential to put LRS technologies centerstage in medical genetics, as reviewed by Logsdon et al. (2020). LRS in combination with linkage analysis has also been successfully applied in a few PGT-M cases (Cheng et al., 2021; Liu et al., 2021). In two studies, LRS analysis for PGT-M was performed directly on embryos (Madjunkova et al., 2020; Zhang et al., 2021). LRS was previously used in prospective parents to characterize breakpoint regions or map SNPs in regions of interest in cases of PGT-M. The embryos were then analyzed either by targeted (Cheng et al., 2021) or genome-wide PGT approaches (Zhang et al., 2019). In this study, we implemented long-read amplicon sequencing as part of a preclinical PGT workup for couples in which partners carry pathogenic variants resulting in cardio-facio-cutaneous syndrome 3, very-long-chain acyl-CoA dehydrogenase deficiency (VLCAD) and b-thalassemia.

High-molecular-weight genomic DNA is needed for the long-read sequencing of three-generation sequencing, but because of concerns about excessive damage to the embryo, the number of cells biopsied at most PGT centers is usually less than 10 cells. Because PGT centers typically prefer to use traditional spin columns to extract genomic DNA, it is difficult to obtain the total amount of 2 µg of DNA needed for three-generation sequencing. In comparison, NGS-based Phbol-seq has more advantages. Simultaneously, Phbol-seq is a growth fragment sequencing method based on linked reads. The sequencing platform is a second-generation sequencing platform, the library construction cost is low, and the main detection cost is the sequencing cost. With the gradual reduction in the detection cost of second-generation sequencing platforms, this method has gradually become accepted by some reproduction centers. Therefore, high-depth sequencing of the whole genome (approximately 30x) can be used to construct a haplotype for all monogenic diseases of chromosomes that can be detected by NGS, and this approach will be used as a universal detection method in the future.

Our data show that Phbol-seq from embryos on TE biopsies could provide an alternative approach to PGT for couples with incomplete families and *de novo* mutations. Logical recombination events within the region of interest reduce the risk of misdiagnosis. Finally, embryos diagnosed as mutation carriers by targeting long-read haplotypes can serve as a stage reference for integrated PGT, providing reliable haplotype and copy number information, as has been recently demonstrated. For suspected gonadal mosaicism, although no successful cases were reported in this paper, for couples whose peripheral blood gene test results were normal but who repeatedly conceived of children with the same genetic disease, according to the general method of haplotype construction, a certain proportion of reproductive mosaicism can be confirmed in the couple’s reproductive cells by preliminary target sequence capture ultradeep sequencing technology. The haplotype was subsequently constructed by the method used in these cases (which may require a greater sequencing depth). For the constructed haplotype, the probability of carrying a reproductive chimeric mutation is 0 for the nonmutated haplotype, while for the haplotype carrying a reproductive chimeric mutation, if the embryo is the carrier type, there is a certain probability that it will carry the mutation. Therefore, completely blocking the risk haplotype is recommended.

Nevertheless, we have demonstrated the reliable accuracy of haplotype staging using long-read amplicon sequencing. Together with the cost per sample, improved data quality and relatively simple workflow similar to any other NGS technique, Phbol-seq long-read amplicon sequencing offers a valuable preclinical PGT screening strategy for couples with neonatal pathogenic variants. Simultaneously, the sequencing platform used was a second-generation sequencing platform. Haplotypes can be constructed as long as single-gene disease mutations and structural variations can be detected by the second-generation sequencing platform. Therefore, there is no need to construct personalized methods for these patients. However, as Phbol-seq is based on NGS sequencing, it has the usual limitations of NGS (Alkan et al., 2011; Daber et al., 2013; Abbasi and Alexandrov, 2021; Zhong et al., 2021). For example, coverage of the centromere and proximal telomere regions is relatively poor, and variation in this part of the region cannot be classified. In addition, there are more common high GC regions, such as the PKD1 exon 1 regions, which are not covered by Phbol-seq, so typing of these sites is not successful. In addition, the regions with true and false genes, such as SMN1/SMN2, have homologous sequence interference of large fragments, and the comparison results are not reliable; therefore, the haplotype cannot be successfully constructed at present. However, for some genes with relatively small homologous regions, such as the IKBKG gene, the true comparison of true and false genes can be made using barcode tag information (Antil et al., 2023). In addition, after DNA molecular tagging, the barcode will have some deletion of some regions, and it is difficult to tag some high GC or repetitive regions (Ring et al., 2018); therefore, these regions may not be made into DNA libraries themselves. Thus, patients should be made aware of the current limitations of the technology and the need for further development to offer universal PGT to couples at high risk of noninformative results.

We believe that with all the advances we have seen in the LRS field, faster, more accurate methods will emerge and potentially streamline the entire PGT process, ultimately eliminating the need for preclinical PGT steps or more family members for PGT patients.

## 8 Conclusion

This study demonstrates the feasibility of long fragment sequencing for the accurate detection of point mutations and small fragment deletions in carriers of monogenic diseases, and in the clinical PGT-M cycle, long fragment sequencing can be used to distinguish whether embryos carry parental pathogenic variants. In this study, current methods for detecting and blocking single-gene diseases caused by point mutations and small fragment deletions were improved and extended by long fragment sequencing technology. The results of Phbol-seq-based PGT-M were accurate and feasible by Sanger sequencing and prenatal diagnosis. The content of this study will help more monogenic disease carriers with incomplete families, *de novo* mutations or suspected germline mosaicism give birth to healthy babies with a normal phenotype.

## Data Availability

The datasets presented in this study can be found in online repositories. The names of the repository/repositories and accession number(s) can be found in the article/Supplementary Material.
