# Supplementary material for: Proband-independent haplotyping based on NGS-based long-read sequencing for detecting pathogenic variant carrier status in preimplantation genetic testing for monogenic diseases
Source: Front Mol Biosci. 2024 Mar 7;11:1329580. doi: 10.3389/fmolb.2024.1329580 (PMC10955336; doi:10.3389/fmolb.2024.1329580)
Supplement: Supplementary file 1 [file DataSheet1.pdf]

Supplementary figure1 Genome-wide scan for fetal amniotic cells

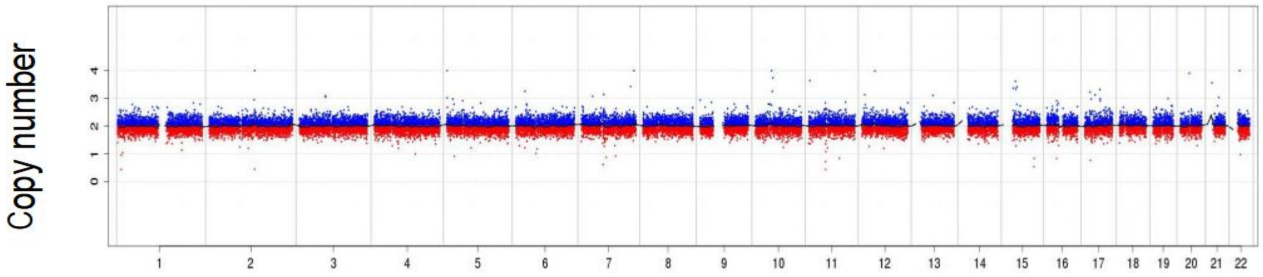

A

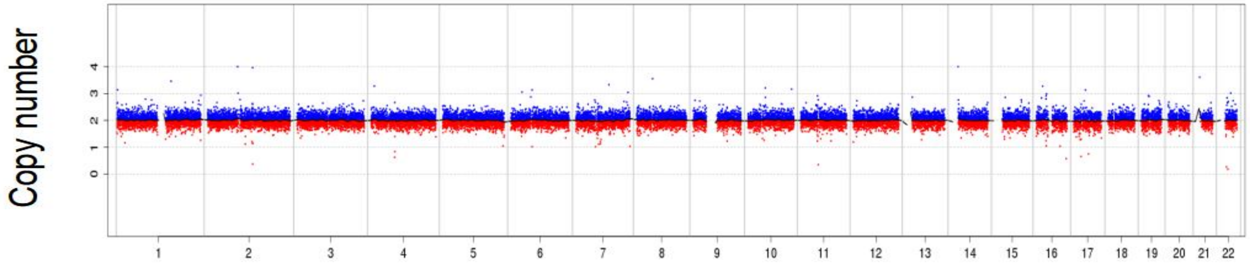

B

supplementary figure 1. Genome-wide scan for fetal amniotic cells. (A) NGS result of fetal amniotic cells shows that the fetus in the family MI is euploid; **(B)** NGS result of fetal amniotic cells shows that the fetus in the family MII is euploid.

Supplementary Table 1.1   Family MI SNP chain analysis results

SNP locus		Female		Male		MI-E1		MI-E2		MI-E3		MI-E4		MI-E5	
		M1	M0	F0	F1	M0	F1	M0	F0	M1	F1	M0	F1	M1	F1
Chr15	64860837	G	G	A	G	G	G	G	A	G	G	G	G	G	G
Chr15	65183801	A	G	G	G	G	G	G	G	A	G	G	G	A	G
Chr15	65183950	A	C	C	C	C	C	C	C	A	C	C	C	A	C
Chr15	65201166	G	A	G	G	A	G	A	G	G	G	A	G	G	G
Chr15	65207670	G	A	A	A	A	A	A	A	G	A	A	A	G	A
Chr15	65231831	A	C	C	C	C	C	C	C	A	C	C	C	A	C
Chr15	65231902	C	T	T	T	T	T	T	T	C	T	T	T	C	T
Chr15	65315961	T	C	C	C	C	C	C	C	T	C	C	C	T	C
Chr15	65505785	C	C	A	C	C	C	C	A	C	C	C	C	C	C
Chr15	65647765	G	G	A	G	G	G	G	A	G	G	G	G	G	G

Chr15	65689267	C	T	T	T	T	T	T	T	C	T	T	T	C	T
Chr15	65695983	A	G	A	A	G	A	G	A	A	A	G	A	A	A
Chr15	66146282	C	C	T	C	C	C	C	T	C	C	C	C	C	C
Chr15	66181936	C	C	A	C	C	C	C	A	C	C	C	C	C	C
Chr15	66199495	T	T	C	T	T	T	T	C	T	T	T	T	T	T
Chr15	66341303	A	C	C	C	C	C	C	C	A	C	C	C	A	C
Chr15	66359605	T	T	T	C	T	C	T	T	T	C	T	C	T	C
Chr15	66423612	T	T	C	T	T	T	T	C	T	T	T	T	T	T
Chr15	66447831	C	C	T	C	C	C	C	T	C	C	C	C	C	C
Chr15	66454686	C	C	T	C	C	C	C	T	C	C	C	C	C	C
Chr15	66466967	T	T	C	T	T	T	T	C	T	T	T	T	T	T
Chr15	66471195	A	C	C	C	C	C	C	C	A	C	C	C	A	C
Chr15	66483378	T	C	T	T	C	T	C	T	T	T	C	T	T	T

Chr15	66503584	A	G	G	G	G	G	G	G	A	G	G	G	A	G
Chr15	66531427	C	T	T	T	T	T	T	T	C	T	T	T	C	T
Chr15	66560723	T	C	C	C	C	C	C	C	T	C	C	C	T	C
Chr15	66562591	C	A	A	A	A	A	A	A	C	A	A	A	C	A
Chr15	66686770	A	G	G	G	G	G	G	G	A	G	G	G	A	G
Chr15	66691474	A	A	G	A	A	A	A	G	A	A	A	A	A	A
				**Female pathogenic variation (Chr15:66727483)**											
Chr15	66821250	G	A	A	A	A	A	A	A	G	A	A	A	G	A
Chr15	66825594	G	G	A	G	G	G	G	A	G	G	G	G	G	G
Chr15	66874870	C	C	C	T	C	T	C	C	C	T	C	T	C	T
Chr15	66930862	T	T	C	T	T	T	T	C	T	T	T	T	T	T
Chr15	66933908	C	T	T	T	T	T	T	T	C	T	T	T	C	T
Chr15	66954426	G	G	G	A	G	A	G	G	G	A	G	A	G	A

Chr15	66960452	C	C	C	T	C	T	C	C	C	T	C	T	C	T
Chr15	66981329	T	C	T	T	C	T	C	T	T	T	C	T	T	T
Chr15	67004334	A	G	A	A	G	A	G	A	A	A	G	A	A	A
Chr15	67012137	C	T	C	C	T	C	T	C	C	C	T	C	C	C
Chr15	67015660	G	A	G	G	A	G	A	G	G	G	A	G	G	G
Chr15	67059259	A	G	A	A	G	A	G	A	A	A	G	A	A	A
Chr15	67066563	C	T	C	C	T	C	T	C	C	C	T	C	C	C
Chr15	67079851	A	G	A	A	G	A	G	A	A	A	G	A	A	A
Chr15	67091032	A	G	G	G	G	G	G	G	A	G	G	G	A	G
Chr15	67101284	C	A	A	A	A	A	A	A	C	A	A	A	C	A
Chr15	67228085	C	C	C	T	C	T	C	C	C	T	C	T	C	T
Chr15	67247554	T	T	T	C	T	C	T	T	T	C	T	C	T	C
Chr15	67252685	A	A	G	A	A	A	A	G	A	A	A	A	A	A

Chr15	67252716	G	G	G	A	G	A	G	G	G	A	G	A	G	A
Chr15	67253823	T	T	C	T	T	T	T	C	T	T	T	T	T	T
Chr15	67257314	G	G	A	G	G	G	G	A	G	G	G	G	G	G
Chr15	67391739	G	A	A	A	A	A	A	A	G	A	A	A	G	A
Chr15	67392656	A	G	G	G	G	G	G	G	A	G	G	G	A	G
Chr15	67442596	T	C	C	C	C	C	C	C	T	C	C	C	T	C
Chr15	67450305	G	A	A	A	A	A	A	A	G	A	A	A	G	A
Chr15	67464291	G	A	A	A	A	A	A	A	G	A	A	A	G	A
Chr15	67468285	G	A	A	A	A	A	A	A	G	A	A	A	G	A
Chr15	67468525	A	G	G	G	G	G	G	G	A	G	G	G	A	G
Chr15	67469118	A	G	G	G	G	G	G	G	A	G	G	G	A	G
Chr15	67675128	C	C	C	A	C	A	C	C	C	A	C	A	C	A
Chr15	67697340	A	A	G	A	A	A	A	G	A	A	A	A	A	A

Chr15	68126327	C	C	C	A	C	A	C	C	C	A	C	A	C	A
Chr15	68140604	C	C	C	A	C	A	C	C	C	A	C	A	C	A
Chr15	68437838	G	G	A	G	G	G	G	A	G	G	G	G	G	G
Chr15	68605840	C	C	T	C	C	C	C	T	C	C	C	C	C	C
Chr15	68608490	T	T	C	T	T	T	T	C	T	T	T	T	T	T
Chr15	68608791	T	T	C	T	T	T	T	C	T	T	T	T	T	T
Chr15	68610551	T	C	T	T	C	T	C	T	T	T	C	T	T	T
Chr15	68611051	G	G	A	G	G	G	G	A	G	G	G	G	G	G
Chr15	68611377	T	T	T	C	T	C	T	T	T	C	T	C	T	C
Chr15	68615519	T	T	C	T	T	T	T	C	T	T	T	T	T	T
Chr15	68628163	C	C	T	C	C	C	C	T	C	C	C	C	C	C
Chr15	68631017	T	C	C	C	C	C	C	C	T	C	C	C	T	C
Chr15	68680125	C	C	C	T	C	T	C	C	C	T	C	T	C	T

Chr15	68698192	C	T	C	C	T	C	T	C	C	C	T	C	C	C
Chr15	68717979	C	T	C	C	T	C	T	C	C	C	T	C	C	C
Chr15	68732250	C	A	C	C	A	C	A	C	C	C	A	C	C	C
Chr15	68779539	C	T	C	C	T	C	T	C	C	C	T	C	C	C
Chr15	68781340	G	A	A	A	A	A	A	A	G	A	A	A	G	A
Chr15	68781680	A	G	G	G	G	G	G	G	A	G	G	G	A	G

Note: The bold red font represents the pathogenic variation. The pink font represents the female valid locus. The yellow font represents the male valid locus. The blue font represents the Chromatid where the female origin mutation locus is located. "M0" represents the female normal Chromatid. "M1" represents the female high-risk Chromatid. "F0、F1" represents the male normal Chromatid. "MISS" represents the missed locus. "/" represents a possible allele drop-out (ADO) or de novo mutation at the locus.

Abbreviations: Chr: Chromosome; SNP: Single Nucleotide Polymorphism.

Supplementary Table 1.2 Family MII SNP chain analysis results

SNP locus		Female		Male		MII-E1		MII-E2		MII-E3		MII-E4		MII-E5		MII-E6		MII-E7		MII-E8		MII-E9	
		M1	M0	F1	F0	M0	F0	M1	F0	M0/ M1	F1	M0/ M1	F1	M0	F1	M1	F1	M1	F1	M0	F0	M1	F1
Chr17	5323125	A	C	A	A	C	A	A	A	C	A	C	A	C	A	A	A	A	A	C	A	A	A
Chr17	5326162	A	G	A	A	G	A	A	A	G	A	G	A	G	A	A	A	A	A	G	A	A	A
Chr17	5326341	T	C	T	T	C	T	T	T	C	T	C	T	C	T	T	T	T	T	C	T	T	T
Chr17	5639077	C	T	C	C	T	C	C	C	T	C	T	C	T	C	C	C	C	C	T	C	C	C
Chr17	5653744	A	A	A	C	A	C	A	C	A	A	A	A	A	A	A	A	A	A	A	C	A	A
Chr17	5687387	G	G	G	A	G	A	G	A	G	G	G	G	G	G	G	G	G	G	G	A	G	G
Chr17	5691873	C	C	C	T	C	T	C	T	C	C	C	C	C	C	C	C	C	C	C	T	C	C
Chr17	5706623	A	A	A	G	A	G	A	G	A	A	A	A	A	A	A	A	A	A	A	G	A	A
Chr17	5723206	A	G	A	A	G	A	A	A	G	A	G	A	G	A	A	A	A	A	G	A	A	A
Chr17	5733558	A	G	A	A	G	A	A	A	G	A	G	A	G	A	A	A	A	A	G	A	A	A
Chr17	5742771	C	T	C	C	T	C	C	C	T	C	T	C	T	C	C	C	C	C	T	C	C	C
Chr17	5749880	A	G	A	A	G	A	A	A	G	A	G	A	G	A	A	A	A	A	G	A	A	A
Chr17	5750188	G	A	G	G	A	G	G	G	A	G	A	G	A	G	G	G	G	G	A	G	G	G
Chr17	5757530	G	A	G	G	A	G	G	G	A	G	A	G	A	G	G	G	G	G	A	G	G	G
Chr17	5760191	A	A	A	G	A	G	A	G	A	A	A	A	A	A	A	A	A	A	A	G	A	A
Chr17	5768989	A	A	A	G	A	G	A	G	A	A	A	A	A	A	A	A	A	A	A	G	A	A
Chr17	5769363	G	G	G	A	G	A	G	A	G	G	G	G	G	G	G	G	G	G	G	A	G	G
Chr17	6007144	A	G	A	A	G	A	A	A	G	A	G	A	G	A	A	A	A	A	G	A	A	A
Chr17	6018493	A	G	A	A	G	A	A	A	G	A	G	A	G	A	A	A	A	A	G	A	A	A
Chr17	6141677	C	A	C	C	A	C	C	C	A	C	A	C	A	C	C	C	C	C	A	C	C	C

Chr17	6285864	G	G	G	A	G	A	G	A	G	G	G	G	G	G	G	G	G	G	G	A	G	G
Chr17	6317990	C	T	C	C	T	C	C	C	T	C	T	C	T	C	C	C	C	C	T	C	C	C
Chr17	6324623	C	T	C	C	T	C	C	C	T	C	T	C	T	C	C	C	C	C	T	C	C	C
Chr17	6409394	C	C	C	A	C	A	C	A	C	C	C	C	C	C	C	C	C	C	C	A	C	C
Chr17	6609596	C	A	C	C	A	C	C	C	A	C	A	C	A	C	C	C	C	C	A	C	C	C
Chr17	6613776	A	C	A	A	C	A	A	A	C	A	C	A	C	A	A	A	A	A	C	A	A	A
Chr17	6636204	A	G	A	A	G	A	A	A	G	A	G	A	G	A	A	A	A	A	G	A	A	A
Chr17	6658367	G	A	G	G	A	G	G	G	A	G	A	G	A	G	G	G	G	G	A	G	G	G
Chr17	6670757	G	A	G	G	A	G	G	G	A	G	A	G	A	G	G	G	G	G	A	G	G	G
Chr17	6727629	G	G	G	A	G	A	G	A	G	G	G	G	G	G	G	G	G	G	G	A	G	G
Chr17	6729550	G	A	G	G	A	G	G	G	A	G	A	G	A	G	G	G	G	G	A	G	G	G
Chr17	6731181	A	A	A	G	A	G	A	G	A	A	A	A	A	A	A	A	A	A	A	G	A	A
Chr17	6850368	T	C	T	T	C	T	T	T	T	T	C	T	C	T	T	T	T	T	C	T	T	T
Chr17	6852586	C	T	C	C	T	C	C	C	C	C	T	C	T	C	C	C	C	C	T	C	C	C
Chr17	6857535	C	T	C	C	T	C	C	C	C	C	T	C	T	C	C	C	C	C	T	C	C	C
Chr17	6880719	G	G	G	A	G	A	G	A	G	G	G	G	G	G	G	G	G	G	G	A	G	G
Chr17	6902760	G	G	G	A	G	A	G	A	G	G	G	G	G	G	G	G	G	G	G	A	G	G
Chr17	6903944	A	A	A	G	A	G	A	G	A	A	A	A	A	A	A	A	A	A	A	G	A	A
Chr17	6969063	C	C	C	T	C	T	C	T	C	C	C	C	C	C	C	C	C	C	C	T	C	C
Chr17	7017189	G	A	G	G	A	G	G	G	G	G	A	G	A	G	G	G	G	G	A	G	G	G
**Female /Male pathogenic variation (Chr17:7127338-7127341)**																							
Chr17	7217340	A	A	A	G	A	G	A	G	A	A	A	A	A	A	A	A	A	A	A	G	A	A
Chr17	7265497	A	G	A	A	G	A	A	A	A	A	G	A	G	A	A	A	A	A	G	A	A	A
Chr17	7290821	T	T	T	C	T	C	T	C	T	T	T	T	T	T	T	T	T	T	T	C	T	T

Chr17	7417663	T	T	T	C	T	C	T	C	T	T	T	T	T	T	T	T	T	T	T	C	T	T
Chr17	7434079	A	A	A	G	A	G	A	G	A	A	A	A	A	A	A	A	A	A	A	G	A	A
Chr17	7440984	T	T	T	C	T	C	T	C	T	T	T	T	T	T	T	T	T	T	T	C	T	T
Chr17	7469229	C	C	C	T	C	T	C	T	C	C	C	C	C	C	C	C	C	C	C	T	C	C
Chr17	7512074	G	G	G	A	G	A	G	A	G	G	G	G	G	G	G	G	G	G	G	A	G	G
Chr17	7530271	C	T	C	C	T	C	C	C	C	C	T	C	T	C	C	C	C	C	T	C	C	C
Chr17	7550553	T	T	T	C	T	C	T	C	T	T	T	T	T	T	T	T	T	T	T	C	T	T
Chr17	7592560	C	T	C	C	T	C	C	C	C	C	T	C	T	C	C	C	C	C	T	C	C	C
Chr17	7619861	T	T	T	C	T	C	T	C	T	T	T	T	T	T	T	T	T	T	T	C	T	T
Chr17	7695477	C	T	C	C	T	C	C	C	C	C	C	C	T	C	C	C	C	C	T	C	C	C
Chr17	7728706	T	C	T	T	C	T	T	T	T	T	T	T	C	T	T	T	T	T	C	T	T	T
Chr17	7961204	C	T	T	T	T	T	C	T	C	T	C	T	T	T	C	T	C	T	T	T	C	T
Chr17	7971267	T	T	T	C	T	C	T	C	T	T	T	T	T	T	T	T	T	T	T	C	T	T
Chr17	8009337	G	T	G	G	T	G	G	G	G	G	G	G	T	G	G	G	G	G	T	G	G	G
Chr17	8014416	A	G	A	A	G	A	A	A	A	A	A	A	G	A	A	A	A	A	G	A	A	A
Chr17	8014563	T	C	T	T	C	T	T	T	T	T	T	T	C	T	T	T	T	T	C	T	T	T
Chr17	8022065	G	G	A	G	G	G	G	G	G	A	G	A	G	A	G	A	G	A	G	G	G	A
Chr17	8038634	G	A	A	A	A	A	G	A	G	A	G	A	A	A	G	A	G	A	A	A	G	A
Chr17	8041014	G	A	A	A	A	A	G	A	G	A	G	A	A	A	G	A	G	A	A	A	G	A
Chr17	8084676	G	A	A	A	A	A	G	A	G	A	G	A	A	A	G	A	G	A	A	A	G	A
Chr17	8167600	T	T	C	T	T	T	T	T	T	C	T	C	T	C	T	C	T	C	T	T	T	C
Chr17	8301642	C	C	T	C	C	C	C	C	C	T	C	T	C	T	C	T	C	T	C	C	C	T
Chr17	8323404	C	T	C	C	T	C	C	C	C	C	C	C	T	C	C	C	C	C	T	C	C	C
Chr17	8414252	A	G	A	A	G	A	A	A	A	A	A	A	G	A	A	A	A	A	G	A	A	A
Chr17	8480178	A	G	A	A	G	A	A	A	A	A	A	A	G	A	A	A	A	A	G	A	A	A

Chr17	8516558	C	A	C	C	A	C	C	C	C	C	C	C	A	C	C	C	C	C	A	C	C	C
Chr17	8552660	T	C	T	T	C	T	T	T	T	T	T	T	C	T	T	T	T	T	C	T	T	T
Chr17	8559766	G	A	G	G	A	G	G	G	G	G	G	G	A	G	G	G	G	G	A	G	G	G
Chr17	8561210	C	T	C	C	T	C	C	C	C	C	C	C	T	C	C	C	C	C	T	C	C	C
Chr17	8611325	G	G	G	A	G	A	G	A	G	G	G	G	G	G	G	G	G	G	G	A	G	G
Chr17	8614578	A	G	A	A	G	A	A	A	A	A	A	A	G	A	A	A	A	A	G	A	A	A
Chr17	8701423	A	G	A	A	G	A	A	A	A	A	A	A	G	A	A	A	A	A	G	A	A	A
Chr17	8703420	C	T	C	C	T	C	C	C	C	C	C	C	T	C	C	C	C	C	T	C	C	C
Chr17	8760133	T	T	C	T	T	T	T	T	T	C	T	C	T	C	T	C	T	C	T	T	T	C
Chr17	8793905	T	T	C	T	T	T	T	T	T	C	T	C	T	C	T	C	T	C	T	T	T	C
Chr17	8800308	C	C	A	C	C	C	C	C	C	A	C	A	C	A	C	A	C	A	C	C	C	A
Chr17	8804067	C	C	T	C	C	C	C	C	C	T	C	T	C	T	C	T	C	T	C	C	C	T
Chr17	8804124	C	C	A	C	C	C	C	C	C	A	C	A	C	A	C	A	C	A	C	C	C	A
Chr17	8879780	T	C	C	C	C	C	T	C	T	C	T	C	C	C	T	C	T	C	C	C	T	C
Chr17	8960883	G	A	G	G	A	G	G	G	G	G	G	G	A	G	G	G	G	G	A	G	G	G
Chr17	9066098	T	C	C	C	C	C	T	C	T	C	T	C	C	C	T	C	T	C	C	C	T	C
Chr17	9066254	C	T	T	T	T	T	C	T	C	T	C	T	T	T	C	T	C	T	T	T	C	T
Chr17	9075977	C	T	C	C	T	C	C	C	C	C	C	C	T	C	C	C	C	C	T	C	C	C
Chr17	9086712	T	C	T	T	C	T	T	T	T	T	T	T	C	T	T	T	T	T	C	T	T	T
Chr17	9089063	C	T	C	C	T	C	C	C	C	C	C	C	T	C	C	C	C	C	T	C	C	C
Chr17	9093265	G	A	G	G	A	G	G	G	G	G	G	G	A	G	G	G	G	G	A	G	G	G
Chr17	9098161	C	T	C	C	T	C	C	C	C	C	C	C	T	C	C	C	C	C	T	C	C	C

Note: The bold red font represents the pathogenic variation.The pink font represents the female valid locus.The yellow font represents the male valid locus. The blue font represents the Chromatid containing the female origin mutation locus. "M0" represents the female normal Chromatid. "M1" represents the female high-risk Chromatid. The green font represents the Chromatid containing the male origin mutation locus. "F0"

represents the male normal Chromatid. "F1" represents the male high-risk Chromatid. "MISS" represents the missed detection locus. "/" represents a possible allele drop-out (ADO) or de novo mutation at the locus.

Abbreviations: Chr: Chromosome; SNP: Single Nucleotide Polymorphism.

Supplementary Table 1.3 Family MIII SNP chain analysis results

SNP locus		Female		Male		MIII-E1		MIII-E2		MIII-E3		MIII-E4		MIII-E5		MIII-E6		MIII-E7		MIII-E8		MIII-E9		MIII-E10	
		M1	M0	F1	F0	M1	F0	M1	F0	M1	F0	M0	F0	M0	F1	M0	F0	M1	F1	M0	F1	M1	F1	M0	F0
Chr11	3253267	G	A	G	G	G	G	G	G	G	G	A	G	A	G	A	G	G	G	A	G	G	G	A	G
Chr11	3266972	T	T	T	C	T	C	T	C	T	C	T	C	T	T	T	C	T	T	T	T	T	T	T	C
Chr11	3364655	C	C	T	C	C	C	C	C	C	C	C	C	C	T	C	C	C	T	C	T	C	T	C	C
Chr11	3368279	A	T	T	T	A	T	A	T	A	T	T	T	T	T	T	T	A	T	T	T	A	T	T	T
Chr11	3395593	G	G	C	G	G	G	G	G	G	G	G	G	G	C	G	G	G	C	G	C	G	C	G	G
Chr11	3396136	C	C	C	T	C	T	C	T	C	T	C	T	C	C	C	T	C	C	C	C	C	C	C	T
Chr11	3399268	A	A	G	A	A	A	A	A	A	A	A	A	A	G	A	A	A	G	A	G	A	G	A	A
Chr11	3399518	G	G	C	G	G	G	G	G	G	G	G	G	G	C	G	G	G	C	G	C	G	C	G	G
Chr11	3399740	G	G	C	G	G	G	G	G	G	G	G	G	G	C	G	G	G	C	G	C	G	C	G	G
Chr11	3439368	G	T	G	G	G	G	G	G	G	G	T	G	T	G	T	G	G	G	T	G	G	G	T	G
Chr11	3439547	T	C	C	C	T	C	T	C	T	C	C	C	C	C	C	C	T	C	C	C	T	C	C	C
Chr11	3439548	G	A	G	G	G	G	G	G	G	G	A	G	A	G	A	G	G	G	A	G	G	G	A	G
Chr11	3445902	C	T	T	T	C	T	C	T	C	T	T	T	T	T	T	T	C	T	T	T	C	T	T	T
Chr11	3446121	A	G	A	A	A	A	A	A	A	A	G	A	G	A	G	A	A	A	G	A	A	A	G	A
Chr11	3447944	C	T	C	C	C	C	C	C	C	C	T	C	T	C	T	C	C	C	T	C	C	C	T	C

Chr11	3447979	T	C	C	C	T	C	T	C	T	C	C	C	C	C	C	C	T	C	C	C	T	C	C	C
Chr11	3448105	C	G	G	G	C	G	C	G	C	G	G	G	G	G	G	G	C	G	G	G	C	G	G	G
Chr11	3448264	C	A	A	A	C	A	C	A	C	A	A	A	A	A	A	A	C	A	A	A	C	A	A	A
Chr11	3450393	T	C	C	C	T	C	T	C	T	C	C	C	C	C	C	C	T	C	C	C	T	C	C	C
Chr11	3456707	T	G	G	G	T	G	T	G	T	G	G	G	G	G	G	G	T	G	G	G	T	G	G	G
Chr11	3460452	T	C	C	C	T	C	T	C	T	C	C	C	C	C	C	C	T	C	C	C	T	C	C	C
Chr11	3468613	G	T	T	T	G	T	G	T	G	T	T	T	T	T	T	T	G	T	T	T	G	T	T	T
Chr11	3468882	G	A	A	A	G	A	G	A	G	A	A	A	A	A	A	A	G	A	A	A	G	A	A	A
Chr11	3470737	A	G	G	G	A	G	A	G	A	G	G	G	G	G	G	G	A	G	G	G	A	G	G	G
Chr11	3478951	C	T	C	C	C	C	C	C	C	C	T	C	T	C	T	C	C	C	T	C	C	C	T	C
Chr11	3478953	G	A	G	G	G	G	G	G	G	G	A	G	A	G	A	G	G	G	A	G	G	G	A	G
Chr11	3479743	A	G	A	A	A	A	A	A	A	A	G	A	G	A	G	A	A	A	G	A	A	A	G	A
Chr11	3480315	T	C	C	C	T	C	T	C	T	C	C	C	C	C	C	C	T	C	C	C	T	C	C	C
Chr11	3480345	C	A	A	A	C	A	C	A	C	A	A	A	A	A	A	A	C	A	A	A	C	A	A	A
Chr11	3481268	T	C	C	C	T	C	T	C	T	C	C	C	C	C	C	C	T	C	C	C	T	C	C	C
Chr11	3481958	C	T	C	C	C	C	C	C	C	C	T	C	T	C	T	C	C	C	T	C	C	C	T	C
Chr11	3482628	T	C	T	T	T	T	T	T	T	T	C	T	C	T	C	T	T	T	C	T	T	T	C	T
Chr11	3482638	A	G	A	A	A	A	A	A	A	A	G	A	G	A	G	A	A	A	G	A	A	A	G	A
Chr11	3483174	C	T	C	C	C	C	C	C	C	C	T	C	T	C	T	C	C	C	T	C	C	C	T	C
Chr11	3483997	G	A	G	G	G	G	G	G	G	G	A	G	A	G	A	G	G	G	A	G	G	G	A	G
Chr11	3485581	A	G	A	A	A	A	A	A	A	A	G	A	G	A	G	A	A	A	G	A	A	A	G	A
Chr11	3490611	C	T	C	C	C	C	C	C	C	C	T	C	T	C	T	C	C	C	T	C	C	C	T	C
Chr11	3497644	C	C	C	T	C	T	C	T	C	T	C	T	C	C	C	T	C	C	C	C	C	C	C	T
Chr11	3509975	G	G	G	A	G	A	G	A	G	A	G	A	G	G	G	A	G	G	G	G	G	G	G	A
Chr11	3511726	A	A	A	G	A	G	A	G	A	G	A	G	A	A	A	G	A	A	A	A	A	A	A	G

Chr11	3512023	T	T	T	C	T	C	T	C	T	C	T	C	T	T	T	C	T	T	T	T	T	T	T	C
Chr11	3532747	T	T	T	C	T	C	T	C	T	C	T	C	T	T	T	C	T	T	T	T	T	T	T	C
Chr11	3532800	C	C	C	T	C	T	C	T	C	T	C	T	C	C	C	T	C	C	C	C	C	C	C	T
Chr11	3532812	T	T	T	C	T	C	T	C	T	C	T	C	T	T	T	C	T	T	T	T	T	T	T	C
Chr11	3533113	T	T	T	G	T	G	T	G	T	G	T	G	T	T	T	G	T	T	T	T	T	T	T	G
Chr11	3533117	C	C	C	T	C	T	C	T	C	T	C	T	C	C	C	T	C	C	C	C	C	C	C	T
Chr11	3541730	C	C	C	T	C	T	C	T	C	T	C	T	C	C	C	T	C	C	C	C	C	C	C	T
Chr11	3547851	G	G	G	A	G	A	G	A	G	A	G	A	G	G	G	A	G	G	G	G	G	G	G	A
Chr11	3556121	C	C	T	C	C	C	C	C	C	C	C	C	C	T	C	C	C	T	C	T	C	T	C	C
Chr11	3560173	C	C	A	C	C	C	C	C	C	C	C	C	C	A	C	C	C	A	C	A	C	A	C	C
Chr11	3568166	A	A	G	A	A	A	A	A	A	A	A	A	A	G	A	A	A	G	A	G	A	G	A	A
Chr11	3568171	C	C	T	C	C	C	C	C	C	C	C	C	C	T	C	C	C	T	C	T	C	T	C	C
Chr11	3568377	C	C	T	C	C	C	C	C	C	C	C	C	C	T	C	C	C	T	C	T	C	T	C	C
Chr11	3569769	A	C	A	A	A	A	A	A	A	A	C	A	C	A	C	A	A	A	C	A	A	A	C	A
Chr11	3571577	C	C	T	C	C	C	C	C	C	C	C	C	C	T	C	C	C	T	C	T	C	T	C	C
Chr11	3576728	C	C	A	C	C	C	C	C	C	C	C	C	C	A	C	C	C	A	C	A	C	A	C	C
Chr11	3578671	A	G	A	A	A	A	A	A	A	A	G	A	G	A	G	A	A	A	G	A	A	A	G	A
Chr11	3581738	G	G	C	G	G	G	G	G	G	G	G	G	G	C	G	G	G	C	G	C	G	C	G	G
Chr11	3589503	C	C	C	T	C	T	C	T	C	T	C	T	C	C	C	T	C	C	C	C	C	C	C	T
Chr11	3589892	T	T	G	T	T	T	T	T	T	T	T	T	T	G	T	T	T	G	T	G	T	G	T	T
Chr11	3590220	C	C	A	C	C	C	C	C	C	C	C	C	C	A	C	C	C	A	C	A	C	A	C	C
Chr11	3597604	C	C	C	A	C	A	C	A	C	A	C	A	C	C	C	A	C	C	C	C	C	C	C	A
Chr11	3597745	G	G	G	A	G	A	G	A	G	A	G	A	G	G	G	A	G	G	G	G	G	G	G	A
Chr11	3604106	C	C	T	C	C	C	C	C	C	C	C	C	C	T	C	C	C	T	C	T	C	T	C	C
Chr11	3607259	C	A	A	A	C	A	C	A	C	A	A	A	A	A	A	A	C	A	A	A	C	A	A	A

Chr11	3607267	C	T	T	T	C	T	C	T	C	T	T	T	T	T	T	T	C	T	T	T	C	T	T	T
Chr11	3613163	G	T	T	T	G	T	G	T	G	T	T	T	T	T	T	T	G	T	T	T	G	T	T	T
Chr11	3614706	G	G	G	A	G	A	G	A	G	A	G	A	G	G	G	A	G	G	G	G	G	G	G	A
Chr11	3618756	A	A	A	G	A	G	A	G	A	G	A	G	A	A	A	G	A	A	A	A	A	A	A	G
Chr11	3619690	T	C	C	C	T	C	T	C	T	C	C	C	C	C	C	C	T	C	C	C	T	C	C	C
Chr11	3626066	A	A	A	C	A	C	A	C	A	C	A	C	A	A	A	C	A	A	A	A	A	A	A	C
Chr11	3626087	G	G	G	A	G	A	G	A	G	A	G	A	G	G	G	A	G	G	G	G	G	G	G	A
Chr11	3626177	C	A	A	A	C	A	C	A	C	A	A	A	A	A	A	A	C	A	A	A	C	A	A	A
Chr11	3627183	G	C	C	C	G	C	G	C	G	C	C	C	C	C	C	C	G	C	C	C	G	C	C	C
Chr11	3634762	A	G	G	G	A	G	A	G	A	G	G	G	G	G	G	G	A	G	G	G	A	G	G	G
Chr11	3647353	A	A	A	T	A	T	A	T	A	T	A	T	A	A	A	T	A	A	A	A	A	A	A	T
Chr11	3649794	A	G	G	G	A	G	A	G	A	G	G	G	G	G	G	G	A	G	G	G	A	G	G	G
Chr11	3681850	G	A	A	A	G	A	G	A	G	A	A	A	A	A	A	A	G	A	A	A	G	A	A	A
Chr11	3706037	T	C	C	C	T	C	T	C	T	C	C	C	C	C	C	C	T	C	C	C	T	C	C	C
Chr11	3709361	G	A	A	A	G	A	G	A	G	A	A	A	A	A	A	A	G	A	A	A	G	A	A	A
Chr11	3715036	G	T	T	T	G	T	G	T	G	T	T	T	T	T	T	T	G	T	T	T	G	T	T	T
Chr11	3737605	T	C	C	C	T	C	T	C	T	C	C	C	C	C	C	C	T	C	C	C	T	C	C	C
Chr11	3748831	G	A	A	A	G	A	G	A	G	A	A	A	A	A	A	A	G	A	A	A	G	A	A	A
Chr11	3749585	C	A	A	A	C	A	C	A	C	A	A	A	A	A	A	A	C	A	A	A	C	A	A	A
Chr11	3768719	T	C	C	C	T	C	T	C	T	C	C	C	C	C	C	C	T	C	C	C	T	C	C	C
Chr11	3777050	T	C	C	C	T	C	T	C	T	C	C	C	C	C	C	C	T	C	C	C	T	C	C	C
Chr11	3778311	C	A	A	A	C	A	C	A	C	A	A	A	A	A	A	A	C	A	A	A	C	A	A	A
Chr11	3778333	C	A	A	A	C	A	C	A	C	A	A	A	A	A	A	A	C	A	A	A	C	A	A	A
Chr11	3778432	C	T	T	T	C	T	C	T	C	T	T	T	T	T	T	T	C	T	T	T	C	T	T	T
Chr11	3795454	C	G	G	G	C	G	C	G	C	G	G	G	G	G	G	G	C	G	G	G	C	G	G	G

Chr11	3795457	G	A	A	A	G	A	G	A	G	A	A	A	A	A	A	A	G	A	A	A	G	A	A	A
Chr11	3798018	G	A	A	A	G	A	G	A	G	A	A	A	A	A	A	A	G	A	A	A	G	A	A	A
Chr11	3802369	C	T	T	T	C	T	C	T	C	T	T	T	T	T	T	T	C	T	T	T	C	T	T	T
Chr11	3805687	T	C	C	C	T	C	T	C	T	C	C	C	C	C	C	C	T	C	C	C	T	C	C	C
Chr11	3812164	C	T	T	T	C	T	C	T	C	T	T	T	T	T	T	T	C	T	T	T	C	T	T	T
Chr11	3812165	A	G	G	G	A	G	A	G	A	G	G	G	G	G	G	G	A	G	G	G	A	G	G	G
Chr11	3812315	C	T	T	T	C	T	C	T	C	T	T	T	T	T	T	T	C	T	T	T	C	T	T	T
Chr11	3853473	T	G	G	G	T	G	T	G	T	G	G	G	G	G	G	G	T	G	G	G	T	G	G	G
Chr11	3854765	G	A	A	A	G	A	G	A	G	A	A	A	A	A	A	A	G	A	A	A	G	A	A	A
Chr11	3857821	C	T	T	T	C	T	C	T	C	T	T	T	T	T	T	T	C	T	T	T	C	T	T	T
Chr11	3862788	T	C	C	C	T	C	T	C	T	C	C	C	C	C	C	C	T	C	C	C	T	C	C	C
Chr11	3879472	T	G	G	G	T	G	T	G	T	G	G	G	G	G	G	G	T	G	G	G	T	G	G	G
Chr11	3879482	T	G	G	G	T	G	T	G	T	G	G	G	G	G	G	G	T	G	G	G	T	G	G	G
Chr11	3921136	G	A	A	A	G	A	G	A	G	A	A	A	A	A	A	A	G	A	A	A	G	A	A	A
Chr11	3930982	G	A	A	A	G	A	G	A	G	A	A	A	A	A	A	A	G	A	A	A	G	A	A	A
Chr11	3937343	C	G	G	G	C	G	C	G	C	G	G	G	G	G	G	G	C	G	G	G	C	G	G	G
Chr11	3950546	C	T	T	T	C	T	C	T	C	T	T	T	T	T	T	T	C	T	T	T	C	T	T	T
Chr11	3954928	C	G	G	G	C	G	C	G	C	G	G	G	G	G	G	G	C	G	G	G	C	G	G	G
Chr11	3955111	G	A	A	A	G	A	G	A	G	A	A	A	A	A	A	A	G	A	A	A	G	A	A	A
Chr11	3956049	A	G	G	G	A	G	A	G	A	G	G	G	G	G	G	G	A	G	G	G	A	G	G	G
Chr11	3958828	T	C	C	C	T	C	T	C	T	C	C	C	C	C	C	C	T	C	C	C	T	C	C	C
Chr11	3974467	T	C	C	C	T	C	T	C	T	C	C	C	C	C	C	C	T	C	C	C	T	C	C	C
Chr11	3977418	T	G	G	G	T	G	T	G	T	G	G	G	G	G	G	G	T	G	G	G	T	G	G	G
Chr11	3982746	T	A	A	A	T	A	T	A	T	A	A	A	A	A	A	A	T	A	A	A	T	A	A	A
Chr11	3999399	G	A	A	A	G	A	G	A	G	A	A	A	A	A	A	A	G	A	A	A	G	A	A	A

Chr11	4001264	T	C	C	C	T	C	T	C	T	C	C	C	C	C	C	C	T	C	C	C	T	C	C	C
Chr11	4013329	T	T	G	T	T	T	T	T	T	T	T	T	T	G	T	T	T	G	T	G	T	G	T	T
Chr11	4045757	G	A	A	A	G	A	G	A	G	A	A	A	A	A	A	A	G	A	A	A	G	A	A	A
Chr11	4046476	G	A	A	A	G	A	G	A	G	A	A	A	A	A	A	A	G	A	A	A	G	A	A	A
Chr11	4055593	T	G	G	G	T	G	T	G	T	G	G	G	G	G	G	G	T	G	G	G	T	G	G	G
Chr11	4070552	G	T	T	T	G	T	G	T	G	T	T	T	T	T	T	T	G	T	T	T	G	T	T	T
Chr11	4071878	A	G	G	G	A	G	A	G	A	G	G	G	G	G	G	G	A	G	G	G	A	G	G	G
Chr11	4090748	G	A	A	A	G	A	G	A	G	A	A	A	A	A	A	A	G	A	A	A	G	A	A	A
Chr11	4102517	T	C	C	C	T	C	T	C	T	C	C	C	C	C	C	C	T	C	C	C	T	C	C	C
Chr11	4104430	A	G	G	G	A	G	A	G	A	G	G	G	G	G	G	G	A	G	G	G	A	G	G	G
Chr11	4115658	T	G	G	G	T	G	T	G	T	G	G	G	G	G	G	G	T	G	G	G	T	G	G	G
Chr11	4120795	T	C	C	C	T	C	T	C	T	C	C	C	C	C	C	C	T	C	C	C	T	C	C	C
Chr11	4120849	C	T	T	T	C	T	C	T	C	T	T	T	T	T	T	T	C	T	T	T	C	T	T	T
Chr11	4123427	C	G	G	G	C	G	C	G	C	G	G	G	G	G	G	G	C	G	G	G	C	G	G	G
Chr11	4125216	T	C	C	C	T	C	T	C	T	C	C	C	C	C	C	C	T	C	C	C	T	C	C	C
Chr11	4126440	A	G	G	G	A	G	A	G	A	G	G	G	G	G	G	G	A	G	G	G	A	G	G	G
Chr11	4129120	C	T	T	T	C	T	C	T	C	T	T	T	T	T	T	T	C	T	T	T	C	T	T	T
Chr11	4132573	A	G	G	G	A	G	A	G	A	G	G	G	G	G	G	G	A	G	G	G	A	G	G	G
Chr11	4137230	G	A	A	A	G	A	G	A	G	A	A	A	A	A	A	A	G	A	A	A	G	A	A	A
Chr11	4145658	A	G	G	G	A	G	A	G	A	G	G	G	G	G	G	G	A	G	G	G	A	G	G	G
Chr11	4152413	C	T	T	T	C	T	C	T	C	T	T	T	T	T	T	T	C	T	T	T	C	T	T	T
Chr11	4156661	G	A	A	A	G	A	G	A	G	A	A	A	A	A	A	A	G	A	A	A	G	A	A	A
Chr11	4162790	T	G	G	G	T	G	T	G	T	G	G	G	G	G	G	G	T	G	G	G	T	G	G	G
Chr11	4163923	A	G	G	G	A	G	A	G	A	G	G	G	G	G	G	G	A	G	G	G	A	G	G	G
Chr11	4164034	C	T	T	T	C	T	C	T	C	T	T	T	T	T	T	T	C	T	T	T	C	T	T	T

Chr11	4167432	A	G	G	G	A	G	A	G	A	G	G	G	G	G	G	G	A	G	G	G	A	G	G	G
Chr11	4167889	A	G	G	G	A	G	A	G	A	G	G	G	G	G	G	G	A	G	G	G	A	G	G	G
Chr11	4167903	A	C	C	C	A	C	A	C	A	C	C	C	C	C	C	C	A	C	C	C	A	C	C	C
Chr11	4169464	C	T	T	T	C	T	C	T	C	T	T	T	T	T	T	T	C	T	T	T	C	T	T	T
Chr11	4206044	G	A	G	G	G	G	G	G	G	G	A	G	A	G	A	G	G	G	A	G	G	G	A	G
Chr11	4343408	C	C	T	C	C	C	C	C	C	C	C	C	C	T	C	C	C	T	C	T	C	T	C	C
Chr11	4344316	T	T	C	T	T	T	T	T	T	T	T	T	T	C	T	T	T	C	T	C	T	C	T	T
Chr11	4365595	G	G	G	C	G	C	G	C	G	C	G	C	G	G	G	C	G	G	G	G	G	G	G	C
Chr11	4376960	T	T	C	T	T	T	T	T	T	T	T	T	T	C	T	T	T	C	T	C	T	C	T	T
Chr11	4394464	T	C	C	C	T	C	T	C	T	C	C	C	C	C	C	C	T	C	C	C	T	C	C	C
Chr11	4396185	G	A	A	A	G	A	G	A	G	A	A	A	A	A	A	A	G	A	A	A	G	A	A	A
Chr11	4403271	C	T	C	C	C	C	C	C	C	C	T	C	T	C	T	C	C	C	T	C	C	C	T	C
Chr11	4404192	A	A	A	G	A	G	A	G	A	G	A	G	A	A	A	G	A	A	A	A	A	A	A	G
Chr11	4419682	G	A	G	G	G	G	G	G	G	G	A	G	A	G	A	G	G	G	A	G	G	G	A	G
Chr11	4427713	T	C	C	C	T	C	T	C	T	C	C	C	C	C	C	C	T	C	C	C	T	C	C	C
Chr11	4434529	C	C	T	C	C	C	C	C	C	C	C	C	C	T	C	C	C	T	C	T	C	T	C	C
Chr11	4442744	G	A	A	A	G	A	G	A	G	A	A	A	A	A	A	A	G	A	A	A	G	A	A	A
Chr11	4468706	C	T	T	T	C	T	C	T	C	T	T	T	T	T	T	T	C	T	T	T	C	T	T	T
Chr11	4468715	G	A	A	A	G	A	G	A	G	A	A	A	A	A	A	A	G	A	A	A	G	A	A	A
Chr11	4477583	T	A	A	A	T	A	T	A	T	A	A	A	A	A	A	A	T	A	A	A	T	A	A	A
Chr11	4478236	A	A	A	C	A	C	A	C	A	C	A	C	A	A	A	C	A	A	A	A	A	A	A	C
Chr11	4478284	A	A	A	C	A	C	A	C	A	C	A	C	A	A	A	C	A	A	A	A	A	A	A	C
Chr11	4484737	G	G	G	A	G	A	G	A	G	A	G	A	G	G	G	A	G	G	G	G	G	G	G	A
Chr11	4496961	T	T	T	C	T	C	T	C	T	C	T	C	T	T	T	C	T	T	T	T	T	T	T	C
Chr11	4498318	C	C	C	T	C	T	C	T	C	T	C	T	C	C	C	T	C	C	C	C	C	C	C	T

Chr11	4519690	C	C	C	T	C	T	C	T	C	T	C	T	C	C	C	T	C	C	C	C	C	C	C	T
Chr11	4528775	A	A	A	G	A	G	A	G	A	G	A	G	A	A	A	G	A	A	A	A	A	A	A	G
Chr11	4535231	C	C	C	T	C	T	C	T	C	T	C	T	C	C	C	T	C	C	C	C	C	C	C	T
Chr11	4535466	G	G	G	T	G	T	G	T	G	T	G	T	G	G	G	T	G	G	G	G	G	G	G	T
Chr11	4535771	C	C	C	T	C	T	C	T	C	T	C	T	C	C	C	T	C	C	C	C	C	C	C	T
Chr11	4536230	C	C	C	T	C	T	C	T	C	T	C	T	C	C	C	T	C	C	C	C	C	C	C	T
Chr11	4537614	C	C	C	G	C	G	C	G	C	G	C	G	C	C	C	G	C	C	C	C	C	C	C	G
Chr11	4539757	T	T	T	C	T	C	T	C	T	C	T	C	T	T	T	C	T	T	T	T	T	T	T	C
Chr11	4540705	T	T	T	G	T	G	T	G	T	G	T	G	T	T	T	G	T	T	T	T	T	T	T	G
Chr11	4546329	G	C	C	C	G	C	G	C	G	C	C	C	C	C	C	C	G	C	C	C	G	C	C	C
Chr11	4546504	A	G	G	G	A	G	A	G	A	G	G	G	G	G	G	G	A	G	G	G	A	G	G	G
Chr11	4549071	A	A	A	G	A	G	A	G	A	G	A	G	A	A	A	G	A	A	A	A	A	A	A	G
Chr11	4555496	C	C	C	A	C	A	C	A	C	A	C	A	C	C	C	A	C	C	C	C	C	C	C	A
Chr11	4559421	A	A	A	G	A	G	A	G	A	G	A	G	A	A	A	G	A	A	A	A	A	A	A	G
Chr11	4559582	C	C	C	A	C	A	C	A	C	A	C	A	C	C	C	A	C	C	C	C	C	C	C	A
Chr11	4561039	T	T	T	C	T	C	T	C	T	C	T	C	T	T	T	C	T	T	T	T	T	T	T	C
Chr11	4561140	G	G	G	T	G	T	G	T	G	T	G	T	G	G	G	T	G	G	G	G	G	G	G	T
Chr11	4561175	A	A	A	C	A	C	A	C	A	C	A	C	A	A	A	C	A	A	A	A	A	A	A	C
Chr11	4562458	T	T	T	A	T	A	T	A	T	A	T	A	T	T	T	A	T	T	T	T	T	T	T	A
Chr11	4565430	A	A	A	G	A	G	A	G	A	G	A	G	A	A	A	G	A	A	A	A	A	A	A	G
Chr11	4565490	G	G	G	A	G	A	G	A	G	A	G	A	G	G	G	A	G	G	G	G	G	G	G	A
Chr11	4566377	C	C	C	T	C	T	C	T	C	T	C	T	C	C	C	T	C	C	C	C	C	C	C	T
Chr11	4570832	G	G	G	T	G	T	G	T	G	T	G	T	G	G	G	T	G	G	G	G	G	G	G	T
Chr11	4574258	C	C	C	A	C	A	C	A	C	A	C	A	C	C	C	A	C	C	C	C	C	C	C	A
Chr11	4576491	A	A	A	G	A	G	A	G	A	G	A	G	A	A	A	G	A	A	A	A	A	A	A	G

Chr11	4578141	C	C	C	G	C	G	C	G	C	G	C	G	C	C	C	G	C	C	C	C	C	C	C	G
Chr11	4578158	C	C	C	G	C	G	C	G	C	G	C	G	C	C	C	G	C	C	C	C	C	C	C	G
Chr11	4660316	C	T	C	C	C	C	C	C	C	C	T	C	T	C	T	C	C	C	T	C	C	C	T	C
Chr11	4665763	T	C	C	C	T	C	T	C	T	C	C	C	C	C	C	C	T	C	C	C	T	C	C	C
Chr11	4666074	C	T	T	T	C	T	C	T	C	T	T	T	T	T	T	T	C	T	T	T	C	T	T	T
Chr11	4666810	A	G	A	A	A	A	A	A	A	A	G	A	G	A	G	A	A	A	G	A	A	A	G	A
Chr11	4675056	G	G	A	G	G	G	G	G	G	G	G	G	G	A	G	G	G	A	G	A	G	A	G	G
Chr11	4678461	C	C	A	C	C	C	C	C	C	C	C	C	C	A	C	C	C	A	C	A	C	A	C	C
Chr11	4678541	A	A	G	A	A	A	A	A	A	A	A	A	A	G	A	A	A	G	A	G	A	G	A	A
Chr11	4679261	G	G	A	G	G	G	G	G	G	G	G	G	G	A	G	G	G	A	G	A	G	A	G	G
Chr11	4679286	T	T	G	T	T	T	T	T	T	T	T	T	T	G	T	T	T	G	T	G	T	G	T	T
Chr11	4687238	T	C	C	C	T	C	T	C	T	C	C	C	C	C	C	C	T	C	C	C	T	C	C	C
Chr11	4687447	A	G	G	G	A	G	A	G	A	G	G	G	G	G	G	G	A	G	G	G	A	G	G	G
Chr11	4688403	T	A	A	A	T	A	T	A	T	A	A	A	A	A	A	A	T	A	A	A	T	A	A	A
Chr11	4688404	G	A	A	A	G	A	G	A	G	A	A	A	A	A	A	A	G	A	A	A	G	A	A	A
Chr11	4694338	G	A	A	A	G	A	G	A	G	A	A	A	A	A	A	A	G	A	A	A	G	A	A	A
Chr11	4696380	A	C	C	C	A	C	A	C	A	C	C	C	C	C	C	C	A	C	C	C	A	C	C	C
Chr11	4696534	A	C	C	C	A	C	A	C	A	C	C	C	C	C	C	C	A	C	C	C	A	C	C	C
Chr11	4702838	G	A	G	G	G	G	G	G	G	G	A	G	A	G	A	G	G	G	A	G	G	G	A	G
Chr11	4725667	C	T	C	C	C	C	C	C	C	C	T	C	T	C	T	C	C	C	T	C	C	C	T	C
Chr11	4729507	C	T	C	C	C	C	C	C	C	C	T	C	T	C	T	C	C	C	T	C	C	C	T	C
Chr11	4729512	T	T	C	T	T	T	T	T	T	T	T	T	T	C	T	T	T	C	T	C	T	C	T	T
Chr11	4729746	A	C	A	A	A	A	A	A	A	A	C	A	C	A	C	A	A	A	C	A	A	A	C	A
Chr11	4757096	T	C	C	C	T	C	T	C	T	C	C	C	C	C	C	C	T	C	C	C	T	C	C	C
Chr11	4806674	G	G	A	G	G	G	G	G	G	G	G	G	G	A	G	G	G	A	G	A	G	A	G	G

Chr11	4813884	C	C	A	C	C	C	C	C	C	C	C	C	C	A	C	C	C	A	C	A	C	A	C	C
Chr11	4813927	T	T	C	T	T	T	T	T	T	T	T	T	T	C	T	T	T	C	T	C	T	C	T	T
Chr11	4814204	A	A	G	A	A	A	A	A	A	A	A	A	A	G	A	A	A	G	A	G	A	G	A	A
Chr11	4814246	G	G	A	G	G	G	G	G	G	G	G	G	G	A	G	G	G	A	G	A	G	A	G	G
Chr11	4833954	A	A	G	A	A	A	A	A	A	A	A	A	A	G	A	A	A	G	A	G	A	G	A	A
Chr11	4839543	A	A	G	A	A	A	A	A	A	A	A	A	A	G	A	A	A	G	A	G	A	G	A	A
Chr11	4840834	T	T	G	T	T	T	T	T	T	T	T	T	T	G	T	T	T	G	T	G	T	G	T	T
Chr11	4937040	T	T	G	T	T	T	T	T	T	T	T	T	T	G	T	T	T	G	T	G	T	G	T	T
Chr11	4937101	G	A	A	A	G	A	G	A	G	A	A	A	A	A	A	A	G	A	A	A	G	A	A	A
Chr11	4937319	T	T	C	T	T	T	T	T	T	T	T	T	T	C	T	T	T	C	T	C	T	C	T	T
Chr11	4951320	A	A	G	A	A	A	A	A	A	A	A	A	A	G	A	A	A	G	A	G	A	G	A	A
Chr11	4951366	G	G	A	G	G	G	G	G	G	G	G	G	G	A	G	G	G	A	G	A	G	A	G	G
Chr11	4951958	T	T	C	T	T	T	T	T	T	T	T	T	T	C	T	T	T	C	T	C	T	C	T	T
Chr11	4984987	C	A	A	A	C	A	C	A	C	A	A	A	A	A	A	A	C	A	A	A	C	A	A	A
Chr11	4986957	C	T	T	T	C	T	C	T	C	T	T	T	T	T	T	T	C	T	T	T	C	T	T	T
Chr11	5006300	T	G	G	G	T	G	T	G	T	G	G	G	G	G	G	G	T	G	G	G	T	G	G	G
Chr11	5015486	C	C	C	T	C	T	C	T	C	T	C	T	C	C	C	T	C	C	C	C	C	C	C	T
Chr11	5016269	G	G	G	A	G	A	G	A	G	A	G	A	G	G	G	A	G	G	G	G	G	G	G	A
Chr11	5016553	A	A	A	G	A	G	A	G	A	G	A	G	A	A	A	G	A	A	A	A	A	A	A	G
Chr11	5016821	G	G	G	A	G	A	G	A	G	A	G	A	G	G	G	A	G	G	G	G	G	G	G	A
Chr11	5020117	C	C	C	G	C	G	C	G	C	G	C	G	C	C	C	G	C	C	C	C	C	C	C	G
Chr11	5020189	C	C	C	T	C	T	C	T	C	T	C	T	C	C	C	T	C	C	C	C	C	C	C	T
Chr11	5022429	T	T	T	C	T	C	T	C	T	C	T	C	T	T	T	C	T	T	T	T	T	T	T	C
Chr11	5031097	G	G	C	G	G	G	G	G	G	G	G	G	G	C	G	G	G	C	G	C	G	C	G	G
Chr11	5034741	C	C	A	C	C	C	C	C	C	C	C	C	C	A	C	C	C	A	C	A	C	A	C	C

Chr11	5037488	A	A	G	A	A	A	A	A	A	A	A	A	A	G	A	A	A	G	A	G	A	G	A	A
Chr11	5038608	A	A	G	A	A	A	A	A	A	A	A	A	A	G	A	A	A	G	A	G	A	G	A	A
Chr11	5048786	T	T	G	T	T	T	T	T	T	T	T	T	T	G	T	T	T	G	T	G	T	G	T	T
Chr11	5048805	G	G	A	G	G	G	G	G	G	G	G	G	G	A	G	G	G	A	G	A	G	A	G	G
Chr11	5056615	A	G	G	G	A	G	A	G	A	G	G	G	G	G	G	G	A	G	G	G	A	G	G	G
Chr11	5056635	G	A	A	A	G	A	G	A	G	A	A	A	A	A	A	A	G	A	A	A	G	A	A	A
Chr11	5056655	C	G	G	G	C	G	C	G	C	G	G	G	G	G	G	G	C	G	G	G	C	G	G	G
Chr11	5059881	G	G	A	G	G	G	G	G	G	G	G	G	G	A	G	G	G	A	G	A	G	A	G	G
Chr11	5061541	G	G	A	G	G	G	G	G	G	G	G	G	G	A	G	G	G	A	G	A	G	A	G	G
Chr11	5061767	G	G	T	G	G	G	G	G	G	G	G	G	G	T	G	G	G	T	G	T	G	T	G	G
Chr11	5062199	G	G	A	G	G	G	G	G	G	G	G	G	G	A	G	G	G	A	G	A	G	A	G	G
Chr11	5062210	A	A	T	A	A	A	A	A	A	A	A	A	A	T	A	A	A	T	A	T	A	T	A	A
Chr11	5064595	A	G	G	G	A	G	A	G	A	G	G	G	G	G	G	G	A	G	G	G	A	G	G	G
Chr11	5064645	C	C	G	C	C	C	C	C	C	C	C	C	C	G	C	C	C	G	C	G	C	G	C	C
Chr11	5067389	T	T	G	T	T	T	T	T	T	T	T	T	T	G	T	T	T	G	T	G	T	G	T	T
Chr11	5067440	G	G	A	G	G	G	G	G	G	G	G	G	G	A	G	G	G	A	G	A	G	A	G	G
Chr11	5067531	G	G	T	G	G	G	G	G	G	G	G	G	G	T	G	G	G	T	G	T	G	T	G	G
Chr11	5067571	G	T	T	T	G	T	G	T	G	T	T	T	T	T	T	T	G	T	T	T	G	T	T	T
Chr11	5075534	A	A	G	A	A	A	A	A	A	A	A	A	A	G	A	A	A	G	A	G	A	G	A	A
Chr11	5075550	G	G	A	G	G	G	G	G	G	G	G	G	G	A	G	G	G	A	G	A	G	A	G	G
Chr11	5075564	C	C	G	C	C	C	C	C	C	C	C	C	C	G	C	C	C	G	C	G	C	G	C	C
Chr11	5076657	A	A	G	A	A	A	A	A	A	A	A	A	A	G	A	A	A	G	A	G	A	G	A	A
Chr11	5076679	A	C	C	C	A	C	A	C	A	C	C	C	C	C	C	C	A	C	C	C	A	C	C	C
Chr11	5081364	T	A	A	A	T	A	T	A	T	A	A	A	A	A	A	A	T	A	A	A	T	A	A	A
Chr11	5081573	T	T	C	T	T	T	T	T	T	T	T	T	T	C	T	T	T	C	T	C	T	C	T	T

Chr11	5090181	G	G	T	G	G	G	G	G	G	G	G	G	G	T	G	G	G	T	G	T	G	T	G	G
Chr11	5107983	A	G	G	G	A	G	A	G	A	G	G	G	G	G	G	G	A	G	G	G	A	G	G	G
Chr11	5107985	A	C	C	C	A	C	A	C	A	C	C	C	C	C	C	C	A	C	C	C	A	C	C	C
Chr11	5110068	A	G	G	G	A	G	A	G	A	G	G	G	G	G	G	G	A	G	G	G	A	G	G	G
Chr11	5124833	A	C	A	A	A	A	A	A	A	A	C	A	C	A	C	A	A	A	C	A	A	A	C	A
Chr11	5125443	G	A	G	G	G	G	G	G	G	G	A	G	A	G	A	G	G	G	A	G	G	G	A	G
Chr11	5134501	T	C	T	T	T	T	T	T	T	T	C	T	C	T	C	T	T	T	C	T	T	T	C	T
Chr11	5141729	G	A	G	G	G	G	G	G	G	G	A	G	A	G	A	G	G	G	A	G	G	G	A	G
Chr11	5185289	G	A	G	G	G	G	G	G	G	G	A	G	A	G	A	G	G	G	A	G	G	G	A	G
Chr11	5187841	C	T	C	C	C	C	C	C	C	C	T	C	T	C	T	C	C	C	T	C	C	C	T	C
Chr11	5187842	T	C	T	T	T	T	T	T	T	T	C	T	C	T	C	T	T	T	C	T	T	T	C	T
Chr11	5188844	C	T	C	C	C	C	C	C	C	C	T	C	T	C	T	C	C	C	T	C	C	C	T	C
Chr11	5191573	G	A	G	G	G	G	G	G	G	G	A	G	A	G	A	G	G	G	A	G	G	G	A	G
Chr11	5191680	G	A	G	G	G	G	G	G	G	G	A	G	A	G	A	G	G	G	A	G	G	G	A	G
Chr11	5193338	C	G	C	C	C	C	C	C	C	C	G	C	G	C	G	C	C	C	G	C	C	C	G	C
Chr11	5193410	T	C	T	T	T	T	T	T	T	T	C	T	C	T	C	T	T	T	C	T	T	T	C	T
Chr11	5194180	A	G	A	A	A	A	A	A	A	A	G	A	G	A	G	A	A	A	G	A	A	A	G	A
Chr11	5200525	C	T	C	C	C	C	C	C	C	C	T	C	T	C	T	C	C	C	T	C	C	C	T	C
Chr11	5200640	T	G	T	T	T	T	T	T	T	T	G	T	G	T	G	T	T	T	G	T	T	T	G	T
Chr11	5203776	A	G	A	A	A	A	A	A	A	A	G	A	G	A	G	A	A	A	G	A	A	A	G	A
Chr11	5204601	C	T	C	C	C	C	C	C	C	C	T	C	T	C	T	C	C	C	T	C	C	C	T	C
Chr11	5204764	C	T	C	C	C	C	C	C	C	C	T	C	T	C	T	C	C	C	T	C	C	C	T	C
Chr11	5204842	C	A	C	C	C	C	C	C	C	C	A	C	A	C	A	C	C	C	A	C	C	C	A	C
Chr11	5209957	C	G	C	C	C	C	C	C	C	C	G	C	G	C	G	C	C	C	G	C	C	C	G	C
Chr11	5209966	C	A	C	C	C	C	C	C	C	C	A	C	A	C	A	C	C	C	A	C	C	C	A	C

Chr11	5210144	T	C	T	T	T	T	T	T	T	T	C	T	C	T	C	T	T	T	C	T	T	T	C	T
Chr11	5212521	T	C	C	C	T	C	T	C	T	C	C	C	C	C	C	C	T	C	C	C	T	C	C	C
Chr11	5212574	T	A	T	T	T	T	T	T	T	T	A	T	A	T	A	T	T	T	A	T	T	T	A	T
Chr11	5212593	A	G	A	A	A	A	A	A	A	A	G	A	G	A	G	A	A	A	G	A	A	A	G	A
Chr11	5233697	T	C	C	C	T	C	T	C	T	C	C	C	C	C	C	C	T	C	C	C	T	C	C	C
Chr11	5234542	C	G	G	G	C	G	C	G	C	G	G	G	G	G	G	G	C	G	G	G	C	G	G	G
Chr11	5234587	T	C	C	C	T	C	T	C	T	C	C	C	C	C	C	C	T	C	C	C	T	C	C	C
Chr11	5236740	G	A	A	A	G	A	G	A	G	A	A	A	A	A	A	A	G	A	A	A	G	A	A	A
Chr11	5237746	C	G	G	G	C	G	C	G	C	G	G	G	G	G	G	G	C	G	G	G	C	G	G	G
Chr11	5241522	T	A	A	A	T	A	T	A	T	A	A	A	A	A	A	A	T	A	A	A	T	A	A	A
Chr11	5244929	C	A	A	A	C	A	C	A	C	A	A	A	A	A	A	A	C	A	A	A	C	A	A	A
Chr11	5246000	T	C	C	C	T	C	T	C	T	C	C	C	C	C	C	C	T	C	C	C	T	C	C	C
**Chr11**	**5247993**	**.**	A	A	A										MISS										
**Chr11**	**5247994**	**.**	A	A	A										MISS										
**Chr11**	**5247995**	**.**	A	A	A										MISS										
**Chr11**	**5247996**	**.**	G	G	G										MISS										
**Chr11**	**5248200**	T	T	**A**	T										MISS										
Chr11	5248842	A	A	G	A	A	A	A	A	A	A	A	A	A	G	A	A	A	G	A	G	A	G	A	A
Chr11	5322101	C	T	C	C	C	C	C	C	C	C	T	C	T	C	T	C	C	C	T	C	C	C	T	C
Chr11	5333954	G	G	G	A	G	A	G	A	G	A	G	A	G	G	G	A	G	G	G	G	G	G	G	A
Chr11	5336884	C	T	C	C	C	C	C	C	C	C	T	C	T	C	T	C	C	C	T	C	C	C	T	C
Chr11	5336973	C	T	C	C	C	C	C	C	C	C	T	C	T	C	T	C	C	C	T	C	C	C	T	C
Chr11	5336998	T	C	T	T	T	T	T	T	T	T	C	T	C	T	C	T	T	T	C	T	T	T	C	T
Chr11	5337008	T	C	T	T	T	T	T	T	T	T	C	T	C	T	C	T	T	T	C	T	T	T	C	T
Chr11	5337022	A	C	A	A	A	A	A	A	A	A	C	A	C	A	C	A	A	A	C	A	A	A	C	A

Chr11	5338375	G	C	G	G	G	G	G	G	G	G	C	G	C	G	C	G	G	G	C	G	G	G	C	G
Chr11	5338439	T	C	T	T	T	T	T	T	T	T	C	T	C	T	C	T	T	T	C	T	T	T	C	T
Chr11	5339195	A	C	A	A	A	A	A	A	A	A	C	A	C	A	C	A	A	A	C	A	A	A	C	A
Chr11	5339312	G	G	A	G	G	G	G	G	G	G	G	G	G	A	G	G	G	A	G	A	G	A	G	G
Chr11	5339350	C	T	C	C	C	C	C	C	C	C	T	C	T	C	T	C	C	C	T	C	C	C	T	C
Chr11	5339892	G	A	G	G	G	G	G	G	G	G	A	G	A	G	A	G	G	G	A	G	G	G	A	G
Chr11	5351572	G	G	G	A	G	A	G	A	G	A	G	A	G	G	G	A	G	G	G	G	G	G	G	A
Chr11	5353394	C	A	A	A	C	A	C	A	C	A	A	A	A	A	A	A	C	A	A	A	C	A	A	A
Chr11	5353500	C	T	T	T	C	T	C	T	C	T	T	T	T	T	T	T	C	T	T	T	C	T	T	T
Chr11	5364742	C	C	C	T	C	T	C	T	C	T	C	T	C	C	C	T	C	C	C	C	C	C	C	T
Chr11	5368635	C	T	T	T	C	T	C	T	C	T	T	T	T	T	T	T	C	T	T	T	C	T	T	T
Chr11	5369725	T	A	A	A	T	A	T	A	T	A	A	A	A	A	A	A	T	A	A	A	T	A	A	A
Chr11	5369776	T	C	C	C	T	C	T	C	T	C	C	C	C	C	C	C	T	C	C	C	T	C	C	C
Chr11	5371325	G	A	A	A	G	A	G	A	G	A	A	A	A	A	A	A	G	A	A	A	G	A	A	A
Chr11	5371372	T	C	C	C	T	C	T	C	T	C	C	C	C	C	C	C	T	C	C	C	T	C	C	C
Chr11	5371463	C	T	T	T	C	T	C	T	C	T	T	T	T	T	T	T	C	T	T	T	C	T	T	T
Chr11	5375763	T	C	C	C	T	C	T	C	T	C	C	C	C	C	C	C	T	C	C	C	T	C	C	C
Chr11	5375792	T	C	C	C	T	C	T	C	T	C	C	C	C	C	C	C	T	C	C	C	T	C	C	C
Chr11	5375819	A	G	G	G	A	G	A	G	A	G	G	G	G	G	G	G	A	G	G	G	A	G	G	G
Chr11	5376798	C	T	T	T	C	T	C	T	C	T	T	T	T	T	T	T	C	T	T	T	C	T	T	T
Chr11	5376810	A	G	G	G	A	G	A	G	A	G	G	G	G	G	G	G	A	G	G	G	A	G	G	G
Chr11	5376812	C	A	A	A	C	A	C	A	C	A	A	A	A	A	A	A	C	A	A	A	C	A	A	A
Chr11	5376856	G	A	A	A	G	A	G	A	G	A	A	A	A	A	A	A	G	A	A	A	G	A	A	A
Chr11	5379097	T	C	C	C	T	C	T	C	T	C	C	C	C	C	C	C	T	C	C	C	T	C	C	C
Chr11	5379105	C	T	T	T	C	T	C	T	C	T	T	T	T	T	T	T	C	T	T	T	C	T	T	T

Chr11	5379243	C	G	G	G	C	G	C	G	C	G	G	G	G	G	G	G	C	G	G	G	C	G	G	G
Chr11	5379258	T	A	A	A	T	A	T	A	T	A	A	A	A	A	A	A	T	A	A	A	T	A	A	A
Chr11	5379750	G	C	C	C	G	C	G	C	G	C	C	C	C	C	C	C	G	C	C	C	G	C	C	C
Chr11	5379952	A	G	G	G	A	G	A	G	A	G	G	G	G	G	G	G	A	G	G	G	A	G	G	G
Chr11	5379968	T	A	A	A	T	A	T	A	T	A	A	A	A	A	A	A	T	A	A	A	T	A	A	A
Chr11	5382386	T	G	G	G	T	G	T	G	T	G	G	G	G	G	G	G	T	G	G	G	T	G	G	G
Chr11	5382390	T	G	G	G	T	G	T	G	T	G	G	G	G	G	G	G	T	G	G	G	T	G	G	G
Chr11	5382591	T	G	G	G	T	G	T	G	T	G	G	G	G	G	G	G	T	G	G	G	T	G	G	G
Chr11	5383015	A	G	G	G	A	G	A	G	A	G	G	G	G	G	G	G	A	G	G	G	A	G	G	G
Chr11	5383798	G	C	C	C	G	C	G	C	G	C	C	C	C	C	C	C	G	C	C	C	G	C	C	C
Chr11	5383823	C	T	T	T	C	T	C	T	C	T	T	T	T	T	T	T	C	T	T	T	C	T	T	T
Chr11	5390150	A	G	G	G	A	G	A	G	A	G	G	G	G	G	G	G	A	G	G	G	A	G	G	G
Chr11	5391164	T	C	C	C	T	C	T	C	T	C	C	C	C	C	C	C	T	C	C	C	T	C	C	C
Chr11	5406314	C	T	T	T	C	T	C	T	C	T	T	T	T	T	T	T	C	T	T	T	C	T	T	T
Chr11	5407522	G	A	A	A	G	A	G	A	G	A	A	A	A	A	A	A	G	A	A	A	G	A	A	A
Chr11	5407525	T	G	G	G	T	G	T	G	T	G	G	G	G	G	G	G	T	G	G	G	T	G	G	G
Chr11	5411239	T	T	T	C	T	C	T	C	T	C	T	C	T	T	T	C	T	T	T	T	T	T	T	C
Chr11	5412038	A	A	A	C	A	C	A	C	A	C	A	C	A	A	A	C	A	A	A	A	A	A	A	C
Chr11	5412059	C	C	C	T	C	T	C	T	C	T	C	T	C	C	C	T	C	C	C	C	C	C	C	T
Chr11	5412598	A	A	A	C	A	C	A	C	A	C	A	C	A	A	A	C	A	A	A	A	A	A	A	C
Chr11	5450387	A	G	G	G	A	G	A	G	A	G	G	G	G	G	G	G	A	G	G	G	A	G	G	G
Chr11	5457264	C	T	T	T	C	T	C	T	C	T	T	T	T	T	T	T	C	T	T	T	C	T	T	T
Chr11	5461031	A	A	A	G	A	G	A	G	A	G	A	G	A	A	A	G	A	A	A	A	A	A	A	G
Chr11	5461263	C	C	C	T	C	T	C	T	C	T	C	T	C	C	C	T	C	C	C	C	C	C	C	T
Chr11	5461297	C	C	C	G	C	G	C	G	C	G	C	G	C	C	C	G	C	C	C	C	C	C	C	G

Chr11	5462905	C	T	T	T	C	T	C	T	C	T	T	T	T	T	T	T	C	T	T	T	C	T	T	T
Chr11	5469576	C	T	T	T	C	T	C	T	C	T	T	T	T	T	T	T	C	T	T	T	C	T	T	T
Chr11	5472371	G	A	A	A	G	A	G	A	G	A	A	A	A	A	A	A	G	A	A	A	G	A	A	A
Chr11	5472735	G	T	T	T	G	T	G	T	G	T	T	T	T	T	T	T	G	T	T	T	G	T	T	T
Chr11	5472767	G	A	A	A	G	A	G	A	G	A	A	A	A	A	A	A	G	A	A	A	G	A	A	A
Chr11	5472768	T	C	C	C	T	C	T	C	T	C	C	C	C	C	C	C	T	C	C	C	T	C	C	C
Chr11	5480902	A	G	A	A	A	A	A	A	A	A	G	A	G	A	G	A	A	A	G	A	A	A	G	A
Chr11	5488282	C	A	A	A	C	A	C	A	C	A	A	A	A	A	A	A	C	A	A	A	C	A	A	A
Chr11	5493370	G	G	A	G	G	G	G	G	G	G	G	G	G	A	G	G	G	A	G	A	G	A	G	G
Chr11	5527164	T	G	G	G	T	G	T	G	T	G	G	G	G	G	G	G	T	G	G	G	T	G	G	G
Chr11	5527218	A	G	G	G	A	G	A	G	A	G	G	G	G	G	G	G	A	G	G	G	A	G	G	G
Chr11	5527265	G	A	A	A	G	A	G	A	G	A	A	A	A	A	A	A	G	A	A	A	G	A	A	A
Chr11	5528177	A	A	C	A	A	A	A	A	A	A	A	A	A	C	A	A	A	C	A	C	A	C	A	A
Chr11	5539670	T	C	C	C	T	C	T	C	T	C	C	C	C	C	C	C	T	C	C	C	T	C	C	C
Chr11	5541179	G	T	T	T	G	T	G	T	G	T	T	T	T	T	T	T	G	T	T	T	G	T	T	T
Chr11	5543803	C	T	T	T	C	T	C	T	C	T	T	T	T	T	T	T	C	T	T	T	C	T	T	T
Chr11	5545271	C	T	T	T	C	T	C	T	C	T	T	T	T	T	T	T	C	T	T	T	C	T	T	T
Chr11	5554182	G	G	G	A	G	A	G	A	G	A	G	A	G	G	G	A	G	G	G	G	G	G	G	A
Chr11	5554286	C	C	C	T	C	T	C	T	C	T	C	T	C	C	C	T	C	C	C	C	C	C	C	T
Chr11	5584996	G	A	A	A	G	A	G	A	G	A	A	A	A	A	A	A	G	A	A	A	G	A	A	A
Chr11	5585352	A	G	G	G	A	G	A	G	A	G	G	G	G	G	G	G	A	G	G	G	A	G	G	G
Chr11	5594093	C	C	C	A	C	A	C	A	C	A	C	A	C	C	C	A	C	C	C	C	C	C	C	A
Chr11	5613668	T	T	T	C	T	C	T	C	T	C	T	C	T	T	T	C	T	T	T	T	T	T	T	C
Chr11	5628260	C	T	T	T	C	T	C	T	C	T	T	T	T	T	T	T	C	T	T	T	C	T	T	T
Chr11	5628302	T	C	C	C	T	C	T	C	T	C	C	C	C	C	C	C	T	C	C	C	T	C	C	C

Chr11	5631972	A	G	G	G	A	G	A	G	A	G	G	G	G	G	G	G	A	G	G	G	A	G	G	G
Chr11	5634045	T	T	A	T	T	T	T	T	T	T	T	T	T	A	T	T	T	A	T	A	T	A	T	T
Chr11	5634255	C	C	T	C	C	C	C	C	C	C	C	C	C	T	C	C	C	T	C	T	C	T	C	C
Chr11	5639873	A	G	G	G	A	G	A	G	A	G	G	G	G	G	G	G	A	G	G	G	A	G	G	G
Chr11	5642001	T	T	C	T	T	T	T	T	T	T	T	T	T	C	T	T	T	C	T	C	T	C	T	T
Chr11	5642026	A	A	T	A	A	A	A	A	A	A	A	A	A	T	A	A	A	T	A	T	A	T	A	A
Chr11	5658187	C	T	T	T	C	T	C	T	C	T	T	T	T	T	T	T	C	T	T	T	C	T	T	T
Chr11	5658204	A	A	G	A	A	A	A	A	A	A	A	A	A	G	A	A	A	G	A	G	A	G	A	A
Chr11	5660458	A	A	G	A	A	A	A	A	A	A	A	A	A	G	A	A	A	G	A	G	A	G	A	A
Chr11	5661508	T	T	C	T	T	T	T	T	T	T	T	T	T	C	T	T	T	C	T	C	T	C	T	T
Chr11	5670610	G	A	A	A	G	A	G	A	G	A	A	A	A	A	A	A	G	A	A	A	G	A	A	A
Chr11	5670671	T	C	C	C	T	C	T	C	T	C	C	C	C	C	C	C	T	C	C	C	T	C	C	C
Chr11	5671511	T	T	T	C	T	C	T	C	T	C	T	C	T	T	T	C	T	T	T	T	T	T	T	C
Chr11	5675972	G	A	A	A	G	A	G	A	G	A	A	A	A	A	A	A	G	A	A	A	G	A	A	A
Chr11	5676149	A	G	G	G	A	G	A	G	A	G	G	G	G	G	G	G	A	G	G	G	A	G	G	G
Chr11	5676383	A	G	G	G	A	G	A	G	A	G	G	G	G	G	G	G	A	G	G	G	A	G	G	G
Chr11	5695858	C	C	G	C	C	C	C	C	C	C	C	C	C	G	C	C	C	G	C	G	C	G	C	C
Chr11	5714200	G	G	T	G	G	G	G	G	G	G	G	G	G	T	G	G	G	T	G	T	G	T	G	G
Chr11	5714230	G	G	A	G	G	G	G	G	G	G	G	G	G	A	G	G	G	A	G	A	G	A	G	G
Chr11	5718817	A	A	G	A	A	A	A	A	A	A	A	A	A	G	A	A	A	G	A	G	A	G	A	A
Chr11	5718975	C	C	A	C	C	C	C	C	C	C	C	C	C	A	C	C	C	A	C	A	C	A	C	C
Chr11	5738242	T	C	C	C	T	C	T	C	T	C	C	C	C	C	C	C	T	C	C	C	T	C	C	C
Chr11	5814145	A	A	T	A	A	A	A	A	A	A	A	A	A	T	A	A	A	T	A	T	A	T	A	A
Chr11	5815137	T	T	A	T	T	T	T	T	T	T	T	T	T	A	T	T	T	A	T	A	T	A	T	T
Chr11	5842909	G	G	A	G	G	G	G	G	G	G	G	G	G	A	G	G	G	A	G	A	G	A	G	G

Chr11	5937377	C	C	C	T	C	T	C	T	C	T	C	T	C	C	C	T	C	C	C	C	C	C	C	T
Chr11	6002798	A	G	G	G	A	G	A	G	A	G	G	G	G	G	G	G	A	G	G	G	A	G	G	G
Chr11	6002820	A	G	G	G	A	G	A	G	A	G	G	G	G	G	G	G	A	G	G	G	A	G	G	G
Chr11	6005759	G	A	A	A	G	A	G	A	G	A	A	A	A	A	A	A	G	A	A	A	G	A	A	A
Chr11	6013332	T	T	C	T	T	T	T	T	T	T	T	T	T	C	T	T	T	C	T	C	T	C	T	T
Chr11	6019186	A	A	G	A	A	A	A	A	A	A	A	A	A	G	A	A	A	G	A	G	A	G	A	A
Chr11	6028268	A	A	G	A	A	A	A	A	A	A	A	A	A	G	A	A	A	G	A	G	A	G	A	A
Chr11	6031941	A	A	T	A	A	A	A	A	A	A	A	A	A	T	A	A	A	T	A	T	A	T	A	A
Chr11	6033593	A	A	G	A	A	A	A	A	A	A	A	A	A	G	A	A	A	G	A	G	A	G	A	A
Chr11	6041609	T	T	A	T	T	T	T	T	T	T	T	T	T	A	T	T	T	A	T	A	T	A	T	T
Chr11	6041763	G	G	A	G	G	G	G	G	G	G	G	G	G	A	G	G	G	A	G	A	G	A	G	G
Chr11	6045931	A	A	G	A	A	A	A	A	A	A	A	A	A	G	A	A	A	G	A	G	A	G	A	A
Chr11	6045952	G	G	C	G	G	G	G	G	G	G	G	G	G	C	G	G	G	C	G	C	G	C	G	G
Chr11	6046675	C	C	G	C	C	C	C	C	C	C	C	C	C	G	C	C	C	G	C	G	C	G	C	C
Chr11	6047905	A	A	A	C	A	C	A	C	A	C	A	C	A	A	A	C	A	A	A	A	A	A	A	C
Chr11	6048056	T	T	C	T	T	T	T	T	T	T	T	T	T	C	T	T	T	C	T	C	T	C	T	T
Chr11	6055869	C	C	A	C	C	C	C	C	C	C	C	C	C	A	C	C	C	A	C	A	C	A	C	C
Chr11	6058391	T	T	C	T	T	T	T	T	T	T	T	T	T	C	T	T	T	C	T	C	T	C	T	T
Chr11	6069067	C	C	G	C	C	C	C	C	C	C	C	C	C	G	C	C	C	G	C	G	C	G	C	C
Chr11	6084034	G	G	T	G	G	G	G	G	G	G	G	G	G	T	G	G	G	T	G	T	G	T	G	G
Chr11	6090262	C	C	A	C	C	C	C	C	C	C	C	C	C	A	C	C	C	A	C	A	C	A	C	C
Chr11	6095632	G	G	A	G	G	G	G	G	G	G	G	G	G	A	G	G	G	A	G	A	G	A	G	G
Chr11	6095639	A	A	G	A	A	A	A	A	A	A	A	A	A	G	A	A	A	G	A	G	A	G	A	A
Chr11	6095671	T	T	C	T	T	T	T	T	T	T	T	T	T	C	T	T	T	C	T	C	T	C	T	T
Chr11	6096797	T	T	C	T	T	T	T	T	T	T	T	T	T	C	T	T	T	C	T	C	T	C	T	T

Chr11	6104391	G	G	A	G	G	G	G	G	G	G	G	G	G	A	G	G	G	A	G	A	G	A	G	G
Chr11	6108961	G	G	C	G	G	G	G	G	G	G	G	G	G	C	G	G	G	C	G	C	G	C	G	G
Chr11	6110240	G	G	A	G	G	G	G	G	G	G	G	G	G	A	G	G	G	A	G	A	G	A	G	G
Chr11	6113816	T	T	G	T	T	T	T	T	T	T	T	T	T	G	T	T	T	G	T	G	T	G	T	T
Chr11	6113830	C	C	T	C	C	C	C	C	C	C	C	C	C	T	C	C	C	T	C	T	C	T	C	C
Chr11	6113842	C	C	T	C	C	C	C	C	C	C	C	C	C	T	C	C	C	T	C	T	C	T	C	C
Chr11	6115474	G	G	A	G	G	G	G	G	G	G	G	G	G	A	G	G	G	A	G	A	G	A	G	G
Chr11	6121077	A	A	G	A	A	A	A	A	A	A	A	A	A	G	A	A	A	G	A	G	A	G	A	A
Chr11	6121135	T	T	C	T	T	T	T	T	T	T	T	T	T	C	T	T	T	C	T	C	T	C	T	T
Chr11	6121618	T	T	C	T	T	T	T	T	T	T	T	T	T	C	T	T	T	C	T	C	T	C	T	T
Chr11	6136252	C	C	T	C	C	C	C	C	C	C	C	C	C	T	C	C	C	T	C	T	C	T	C	C
Chr11	6136270	C	C	T	C	C	C	C	C	C	C	C	C	C	T	C	C	C	T	C	T	C	T	C	C
Chr11	6142522	A	A	G	A	A	A	A	A	A	A	A	A	A	G	A	A	A	G	A	G	A	G	A	A
Chr11	6173364	A	A	T	A	A	A	A	A	A	A	A	A	A	T	A	A	A	T	A	T	A	T	A	A
Chr11	6181236	C	C	T	C	C	C	C	C	C	C	C	C	C	T	C	C	C	T	C	T	C	T	C	C
Chr11	6202477	C	T	T	T	C	T	C	T	C	T	T	T	T	T	T	T	C	T	T	T	C	T	T	T
Chr11	6214625	T	C	C	C	T	C	T	C	T	C	C	C	C	C	C	C	T	C	C	C	T	C	C	C
Chr11	6219620	T	C	C	C	T	C	T	C	T	C	C	C	C	C	C	C	T	C	C	C	T	C	C	C
Chr11	6297696	C	C	C	A	C	A	C	A	C	A	C	A	C	C	C	A	C	C	C	C	C	C	C	A
Chr11	6298702	G	G	G	A	G	A	G	A	G	A	G	A	G	G	G	A	G	G	G	G	G	G	G	A
Chr11	6300016	C	C	C	T	C	T	C	T	C	T	C	T	C	C	C	T	C	C	C	C	C	C	C	T
Chr11	6301016	C	T	C	C	C	C	C	C	C	C	T	C	T	C	T	C	C	C	T	C	C	C	T	C
Chr11	6314015	C	T	C	C	C	C	C	C	C	C	T	C	T	C	T	C	C	C	T	C	C	C	T	C
Chr11	6317960	G	A	G	G	G	G	G	G	G	G	A	G	A	G	A	G	G	G	A	G	G	G	A	G
Chr11	6340362	C	C	C	T	C	T	C	T	C	T	C	T	C	C	C	T	C	C	C	C	C	C	C	T

Chr11	6340525	T	A	T	T	T	T	T	T	T	T	A	T	A	T	A	T	T	T	A	T	T	T	A	T
Chr11	6341748	T	C	T	T	T	T	T	T	T	T	C	T	C	T	C	T	T	T	C	T	T	T	C	T
Chr11	6345834	A	G	A	A	A	A	A	A	A	A	G	A	G	A	G	A	A	A	G	A	A	A	G	A
Chr11	6370662	T	T	T	C	T	C	T	C	T	C	T	C	T	T	T	C	T	T	T	T	T	T	T	C
Chr11	6403540	C	T	C	C	C	C	C	C	C	C	T	C	T	C	T	C	C	C	T	C	C	C	T	C
Chr11	6422834	A	G	G	G	A	G	A	G	A	G	G	G	G	G	G	G	A	G	G	G	A	G	G	G
Chr11	6526074	T	C	C	C	T	C	T	C	T	C	C	C	C	C	C	C	T	C	C	C	T	C	C	C
Chr11	6526488	T	T	A	T	T	T	T	T	T	T	T	T	T	A	T	T	T	A	T	A	T	A	T	T
Chr11	6549048	G	G	A	G	G	G	G	G	G	G	G	G	G	A	G	G	G	A	G	A	G	A	G	G
Chr11	6575010	T	T	T	G	T	G	T	G	T	G	T	G	T	T	T	G	T	T	T	T	T	T	T	G
Chr11	6622512	G	G	A	G	G	G	G	G	G	G	G	G	G	A	G	G	G	A	G	A	G	A	G	G
Chr11	6647931	C	C	C	T	C	T	C	T	C	T	C	T	C	C	C	T	C	C	C	C	C	C	C	T
Chr11	6672995	T	C	C	C	T	C	T	C	T	C	C	C	C	C	C	C	T	C	C	C	T	C	C	C
Chr11	6673013	T	T	G	T	T	T	T	T	T	T	T	T	T	G	T	T	T	G	T	G	T	G	T	T
Chr11	6683890	T	A	A	A	T	A	T	A	T	A	A	A	A	A	A	A	T	A	A	A	T	A	A	A
Chr11	6717608	G	T	G	G	G	G	G	G	G	G	T	G	T	G	T	G	G	G	T	G	G	G	T	G
Chr11	6717724	A	C	A	A	A	A	A	A	A	A	C	A	C	A	C	A	A	A	C	A	A	A	C	A
Chr11	6721639	C	T	C	C	C	C	C	C	C	C	T	C	T	C	T	C	C	C	T	C	C	C	T	C
Chr11	6721655	C	T	C	C	C	C	C	C	C	C	T	C	T	C	T	C	C	C	T	C	C	C	T	C
Chr11	6724865	C	T	C	C	C	C	C	C	C	C	T	C	T	C	T	C	C	C	T	C	C	C	T	C
Chr11	6727720	G	A	G	G	G	G	G	G	G	G	A	G	A	G	A	G	G	G	A	G	G	G	A	G
Chr11	6727721	C	G	C	C	C	C	C	C	C	C	G	C	G	C	G	C	C	C	G	C	C	C	G	C
Chr11	6729061	T	C	T	T	T	T	T	T	T	T	C	T	C	T	C	T	T	T	C	T	T	T	C	T
Chr11	6796961	C	C	T	C	C	C	C	C	C	C	C	C	C	T	C	C	C	T	C	T	C	T	C	C
Chr11	6796966	A	G	G	G	A	G	A	G	A	G	G	G	G	G	G	G	A	G	G	G	A	G	G	G

Chr11	6881853	G	T	T	T	G	T	G	T	G	T	T	T	T	T	T	T	G	T	T	T	G	T	T	T
Chr11	6921250	T	T	T	C	T	C	T	C	T	C	T	C	T	T	T	C	T	T	T	T	T	T	T	C
Chr11	6922755	C	C	G	C	C	C	C	C	C	C	C	C	C	G	C	C	C	G	C	G	C	G	C	C
Chr11	6928711	G	G	A	G	G	G	G	G	G	G	G	G	G	A	G	G	G	A	G	A	G	A	G	G
Chr11	6933318	C	C	T	C	C	C	C	C	C	C	C	C	C	T	C	C	C	T	C	T	C	T	C	C
Chr11	6942628	A	A	C	A	A	A	A	A	A	A	A	A	A	C	A	A	A	C	A	C	A	C	A	A
Chr11	6943795	G	G	A	G	G	G	G	G	G	G	G	G	G	A	G	G	G	A	G	A	G	A	G	G
Chr11	6943807	A	A	G	A	A	A	A	A	A	A	A	A	A	G	A	A	A	G	A	G	A	G	A	A
Chr11	6947761	A	A	T	A	A	A	A	A	A	A	A	A	A	T	A	A	A	T	A	T	A	T	A	A
Chr11	6947965	G	G	A	G	G	G	G	G	G	G	G	G	G	A	G	G	G	A	G	A	G	A	G	G
Chr11	6976996	C	T	C	C	C	C	C	C	C	C	T	C	T	C	T	C	C	C	T	C	C	C	T	C
Chr11	7043362	T	T	C	T	T	T	T	T	T	T	T	T	T	C	T	T	T	C	T	C	T	C	T	T
Chr11	7043949	C	C	T	C	C	C	C	C	C	C	C	C	C	T	C	C	C	T	C	T	C	T	C	C
Chr11	7044206	A	A	G	A	A	A	A	A	A	A	A	A	A	G	A	A	A	G	A	G	A	G	A	A
Chr11	7060670	A	A	T	A	A	A	A	A	A	A	A	A	A	T	A	A	A	T	A	T	A	T	A	A
Chr11	7122439	A	G	A	A	A	A	A	A	A	A	G	A	G	A	G	A	A	A	G	A	A	A	G	A
Chr11	7156132	T	T	G	T	T	T	T	T	T	T	T	T	T	G	T	T	T	G	T	G	T	G	T	T
Chr11	7178278	A	T	A	A	A	A	A	A	A	A	T	A	T	A	T	A	A	A	T	A	A	A	T	A
Chr11	7185842	A	T	A	A	A	A	A	A	A	A	T	A	T	A	T	A	A	A	T	A	A	A	T	A
Chr11	7189832	A	G	A	A	A	A	A	A	A	A	G	A	G	A	G	A	A	A	G	A	A	A	G	A
Chr11	7192075	A	C	A	A	A	A	A	A	A	A	C	A	C	A	C	A	A	A	C	A	A	A	C	A
Chr11	7198054	T	G	T	T	T	T	T	T	T	T	G	T	G	T	G	T	T	T	G	T	T	T	G	T
Chr11	7236253	A	G	G	G	A	G	A	G	A	G	G	G	G	G	G	G	A	G	G	G	A	G	G	G

Note: The bold red font represents the pathogenic variation.The pink font represents the female valid locus.The yellow font represents the male valid locus. The blue font represents the Chromatid containing the female origin mutation locus. "M0" represents the female normal Chromatid.

"M1" represents the female high-risk Chromatid. The green font represents the Chromatid containing the male origin mutation locus. "F0" represents the male normal Chromatid. "F1" represents the male high-risk Chromatid. "MISS" represents the missed detection locus. "/" represents a possible allele drop-out (ADO) or de novo mutation at the locus.

Abbreviations: Chr: Chromosome; SNP: Single Nucleotide Polymorphism.
